# The Potential of Traditional Knowledge to Develop Effective Medicines for the Treatment of Leishmaniasis

**DOI:** 10.3389/fphar.2021.690432

**Published:** 2021-06-08

**Authors:** Luiz Felipe D. Passero, Erika dos Santos Brunelli, Thamara Sauini, Thais Fernanda Amorim Pavani, Jéssica Adriana Jesus, Eliana Rodrigues

**Affiliations:** ^1^ Institute of Biosciences, São Paulo State University (UNESP), São Paulo, Brazil; ^2^ Institute for Advanced Studies of Ocean, São Paulo State University (UNESP), São Paulo, Brazil; ^3^ Center for Ethnobotanical and Ethnopharmacological Studies (CEE), Universidade Federal de São Paulo (UNIFESP), São Paulo, Brazil; ^4^ Chemical and Pharmaceutical Research Group (GPQFfesp), Department of Pharmaceutical Sciences, Institute of Environmental, Chemical and Pharmaceutical Sciences, Universidade Federal de São Paulo (UNIFESP), São Paulo, Brazil; ^5^ Laboratório de Patologia de Moléstias Infecciosas (LIM50), Departamento de Patologia, Faculdade de Medicina, Universidade de São Paulo, São Paulo, Brazil

**Keywords:** ethnopharmacology, traditional knowledge, natural drugs, leishmaniasis, medicinal plants, neglected disease

## Abstract

Leishmaniasis is a neglected tropical disease that affects people living in tropical and subtropical areas of the world. There are few therapeutic options for treating this infectious disease, and available drugs induce severe side effects in patients. Different communities have limited access to hospital facilities, as well as classical treatment of leishmaniasis; therefore, they use local natural products as alternative medicines to treat this infectious disease. The present work performed a bibliographic survey worldwide to record plants used by traditional communities to treat leishmaniasis, as well as the uses and peculiarities associated with each plant, which can guide future studies regarding the characterization of new drugs to treat leishmaniasis. A bibliographic survey performed in the *PubMed* and *Scopus* databases retrieved 294 articles related to traditional knowledge, medicinal plants and leishmaniasis; however, only 20 were selected based on the traditional use of plants to treat leishmaniasis. Considering such studies, 378 quotes referring to 292 plants (216 species and 76 genera) that have been used to treat leishmaniasis were recorded, which could be grouped into 89 different families. A broad discussion has been presented regarding the most frequent families, including Fabaceae (27 quotes), Araceae (23), Solanaceae and Asteraceae (22 each). Among the available data in the 378 quotes, it was observed that the parts of the plants most frequently used in local medicine were leaves (42.3% of recipes), applied topically (74.6%) and fresh poultices (17.2%). The contribution of Latin America to studies enrolling ethnopharmacological indications to treat leishmaniasis was evident. Of the 292 plants registered, 79 were tested against *Leishmania* sp. Future studies on leishmanicidal activity could be guided by the 292 plants presented in this study, mainly the five species *Carica papaya* L. (Caricaceae), *Cedrela odorata* L. (Meliaceae), *Copaifera paupera* (Herzog) Dwyer (Fabaceae), *Musa × paradisiaca* L. (Musaceae), and *Nicotiana tabacum* L. (Solanaceae), since they are the most frequently cited in articles and by traditional communities.

## Introduction

The use of plants based on existing empirical knowledge, consecrated by continuous use in traditional communities, directs research, saves time and money in pharmacological and phytochemical studies (Mukherjee et al., 2017). The selection of plants for research and production of drugs, based on claims made by traditional communities regarding a given therapeutic effect in humans, can be a valuable shortcut for the discovery of new active molecules (Süntar, 2020) and to provide, from the academic point of view, evidence for the use of plants as medicines.

Some interesting examples of drugs extracted from plants used in traditional knowledge are (i) alpha humulene from *Varronia curassavica* (Jacq.), which has been used as a topical anti-inflammatory agent (Marques et al., 2019); (ii) quinine, which was purified from *Cinchona* sp. and has antimalarial activity (Boratyński et al., 2019); (iii) galegine from *Galega officinalis* L., which was used as a molecular prototype to synthesize the antidiabetic drug metformin (Bailey, 2017); (iv) morphine and codeine, as hypnoanalgesics, both extracted from *Papaver somniferum* (Stefano et al., 2017); (v) taxol, an antitumour agent extracted from *Taxus brevifolia* Nutt. (Yang and Horwitz, 2017); (vi) vimblastine, an antineoplastic agent, from *Catharanthis roseus* (L.) G. Don (Haque et al., 2018); and (vii) digoxin, purified from Digitalis lanata Ehrh. that displays cardiotonic effect (Patocka et al., 2020), among other examples.

Considering that ethnopharmacological studies have guided the characterization of biologically active molecules and drugs for different diseases, it is evident that this science can contribute to the search for active substances to treat neglected diseases, such as leishmaniasis, an infectious disease caused by parasitic protozoa of the genus *Leishmania*, endemic in tropical and subtropical countries. This neglected infectious disease is transmitted during the blood meal of sandflies of the genera *Lutzomyia* and *Phlebotomus* (Francesquini et al., 2014; Courtenay et al., 2017).

Leishmaniasis has a wide variety of clinical manifestations, from cutaneous to visceral forms (Burza et al., 2018). In cutaneous leishmaniasis (CL), the parasite infects phagocytic cells (mainly macrophages) in the skin tissue. This clinical form is characterized by skin lesions that can be single, multiple or diffuse throughout the body (Gabriel et al., 2019). Some patients have lesions in the mucous membranes, mainly in the upper airways; such injuries can occur years after the resolution of skin lesions (Kevric et al., 2015). Visceral leishmaniasis (VL) is a zoonosis of chronic evolution with systemic involvement. In this clinical form, the parasite migrates to the viscera and infects macrophages in the spleen, liver, lymph nodes, and bone marrow. Typical manifestations are chronic fever, weight loss and hepatosplenomegaly, which can lead to patient death if not properly treated (Hermida et al., 2018). These clinical changes progress along with physiological and histological modifications mainly in the spleen, liver, and bone marrow (Faleiro et al., 2014).

According to the World Health Organization, it is estimated that 50,000 to 90,000 new cases of VL and between 600,00 and one million new cases of CL occur annually. The growth in the number of cases in recent decades has been associated with environmental changes, such as deforestation, irrigation schemes, building dams and urbanization (World Health Organization, 2019). Despite these epidemiological data and the fact that there are different species of parasites occurring in 98 countries, the treatment of this important infectious disease has serious limitations and is based on few drugs, such as pentavalent antimonials, amphotericin B and miltefosine (Passero et al., 2018). Additionally, these drugs induce severe side effects in humans, and in some situations, as is the case of liposomal amphotericin B, high costs limit their use in low-income countries. Furthermore, some species of parasites have become resistant to drugs (Ghorbani and Farhoudi, 2017; Ponte-Sucre et al., 2017).

Considering the epidemiology of leishmaniasis, the scarcity of treatment and the severe side effects of drugs currently used it becomes urgent to find new molecules with leishmanicidal activity. The secondary metabolism of plants offers a panel of molecules with important pharmacological activity, and in leishmaniasis, a series of molecules has already been described with leishmanicidal potential (Passero et al., 2014; Jesus et al., 2017). In this regard, it has been observed that some studies have used the information available in published works about traditional knowledge to select plants, purify bioactive molecules and perform *in vivo* studies; however, only a few works have investigated the natural resources that traditional communities use to treat leishmaniasis and molecules *in vitro* and in *in vivo* models.

Thus, this review intends to investigate, through a bibliographic survey, information about medicinal plants indicated by traditional communities that are employed in the treatment of leishmaniasis, as well as their uses and peculiarities, guiding future studies on the characterization of new compounds with leishmanicidal activity.

## Bibliographic Survey

To verify the existence of scientific studies about plants used by traditional communities to treat leishmaniasis, a bibliographic survey was carried out. For this purpose, a Boolean search was performed in the *Scopus* and *PubMed* databases. It was performed from May to June 2020, and the combination of words was used to expand the possibility of finding data that would meet the expectations of the present study: “(ethnomedicine OR ethnopharmaco* OR ethnobotanic* OR "traditional knowledge") AND (plant OR vegetal) AND (leishmani* OR antileishmani*)”.

The searches in *the PubMed* and *Scopus* databases retrieved a total of 238 and 161 articles, respectively. Additionally, it was observed that 105 articles were common to both databases; therefore, a total of 294 articles were analysed herein. The following exclusion criteria were used in this review: 1) review articles; 2) articles that did not clearly mention the genera or species of studied plants; and 3) articles that demonstrated leishmanicidal activity of plants without having carried out an ethnopharmacological study. The following inclusion criteria were used: 1) original articles from any year, referring to any country; 2) articles that contained clear information about the collection of ethnopharmacological data, except for the literature review; and 3) articles in English, Spanish, Portuguese and French. By considering all of these items, 20 articles were selected and analysed.

**FIGURE 1:**
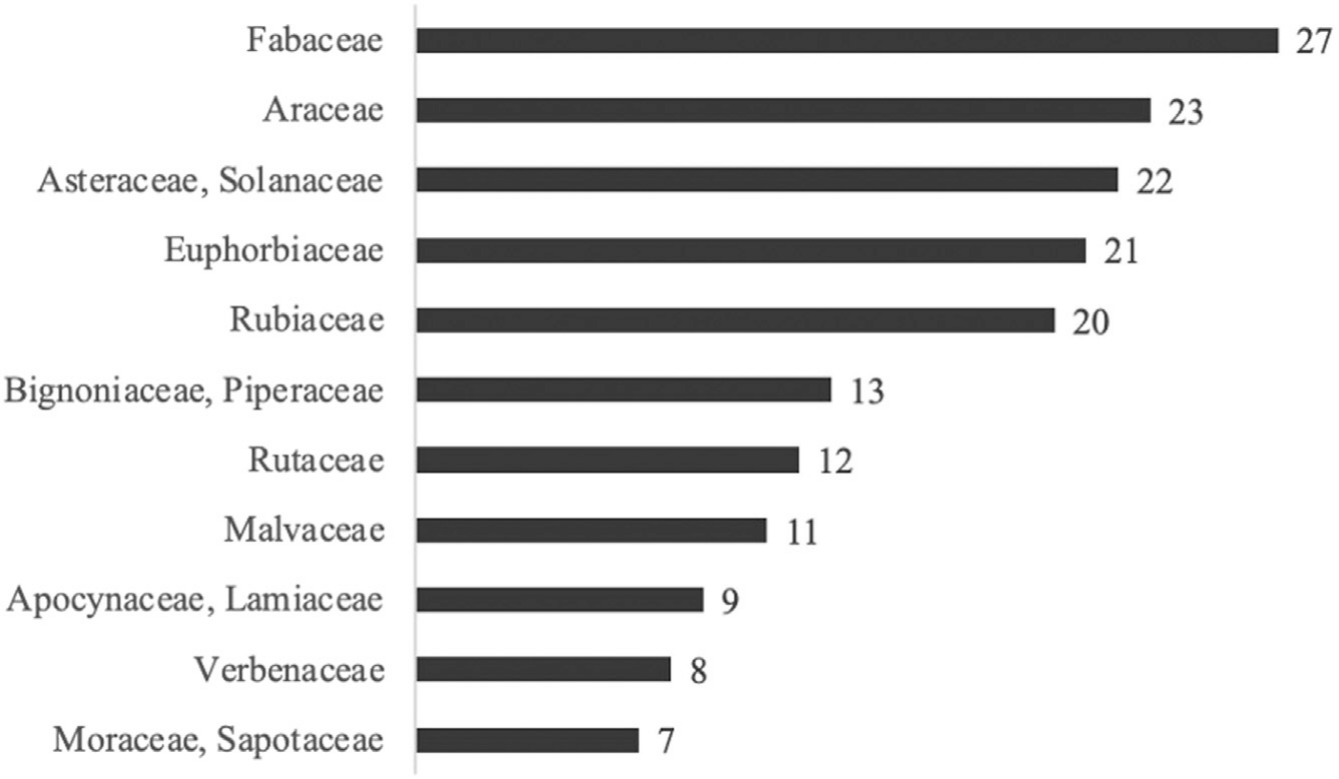
Frequency of the most cited families referring to the 378 quotes of species extracted from the 20 articles, only those species quoted at least 7 times.

**FIGURE 2:**
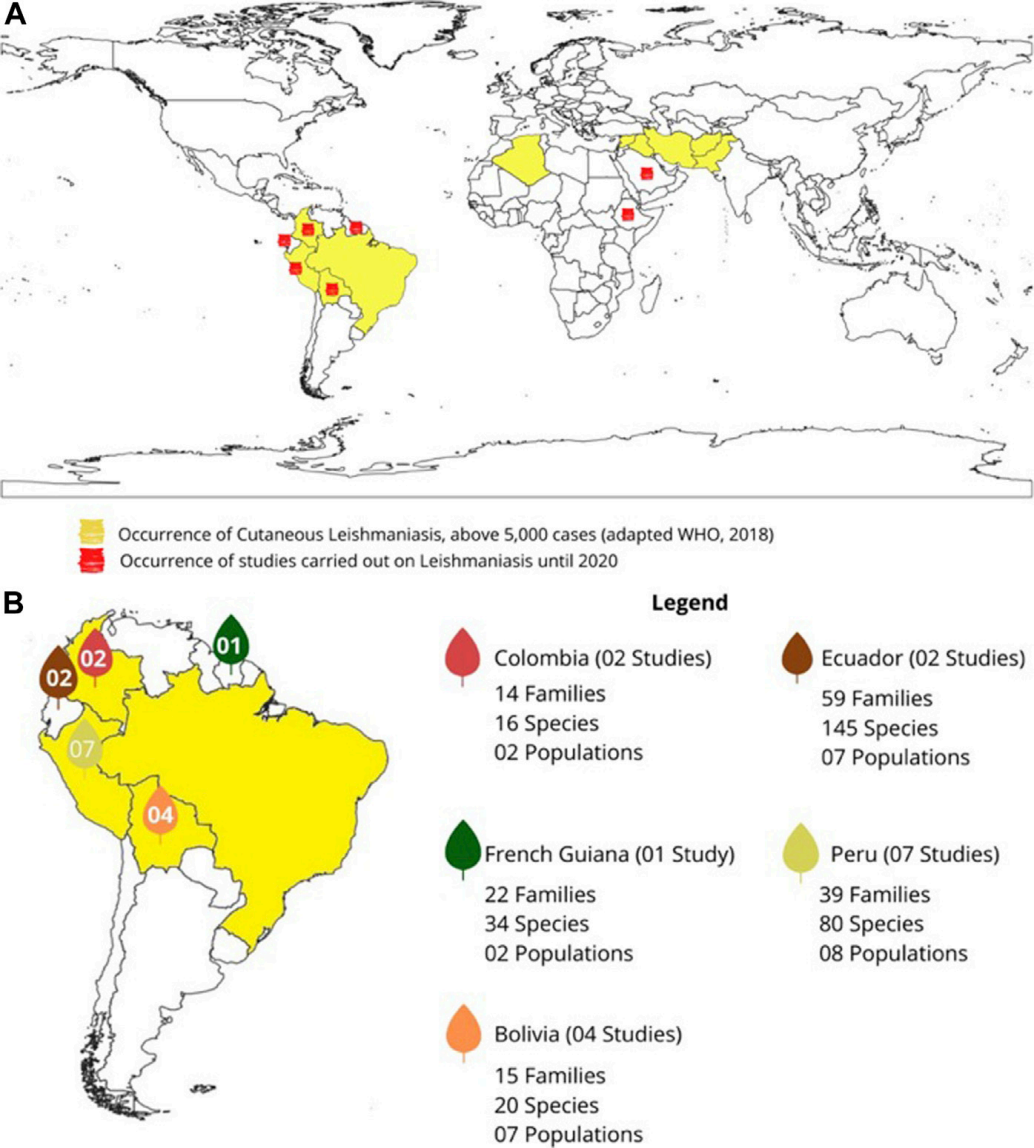
**(A)** World map of the occurrence of cutaneous leishmaniasis and the countries where they have had studies on leishmaniasis. **(B)** Highlights of studies on leishmaniasis carried out in South America.

Plants with identification up to the genus level were included in the present survey, as they represent approximately 20.4% of the total indications. Species indicated with "cf"—whose taxonomic identification could not be confirmed—were also included in the present survey. In addition, all species underwent a review of their correct spelling and current taxonomic classification on the website Plants of the World *online*: http://www.plantsoftheworldonline.org. The following species: *Anthurium muyunense* Croat, *Trema integerrima* (Beurl.) Standl., *Inga bourgonii* (Aubl.) DC., *Meteoridium* sp., and *Citrus aurantiaca* (L.) Swingle, were not found in this website, but data about them were available in the website of TROPICOS: https://www.tropicos.org/home. Species with divergent scientific names in articles and on the website were synonymous, and thus, they were recorded only once. Considering the data found in the selected articles, Tables 1 and 2 and Figures 1 and 2 were included.

**TABLE 1:** The 378 plant quotes obtained from the 20 publications, their families, species/genera, vernacular names, traditional recipes, countries (traditional community), traditional uses, and plants tested for leishmaniasis (*in vitro*).

*Family (Number of quotes and species)* *	Species	Vernacular name	Traditional recipe (plant part, route)	Country (traditional community)	Traditional use (emic term)	Tested for leishmaniasis (results)	References
Acanthaceae (4 quotes and 4 species)	*Fittonia* *sp*.	-	(le, to)	Ecuador#	-	-	Gachet et al. (2010)
	*Hygrophila costata* Nees	Chupador	(ae, to)	Colombia (Afro-Colombian and indigenous groups)		^p^ *La +*	Weniger et al. (2001)
						^p^ *Lb -* ^p^ *Li +*	
						^*a*^ *Lp +*	
	*Hygrophila* *sp*.	-	(wp, to)	Ecuador#		-	Gachet et al. (2010)
	*Sanchezia* *sp*.		(le, to)				
Amaranthaceae (6 quotes and 5 species)	*Alternanthera* *sp*.	-	(le/st, to)	Ecuador#	-	-	Gachet et al. (2010)
	*Amaranthus caudatus* L.	Sangorache	(le)	Ecuador	Cutaneous leishmaniasis		Weigel et al. (1994)
	*Chenopodiastrum murale* (L.) S.Fuentes, Uotila & Borsch	A’Tra	Fresh-po (ap,to)	Saudi Arabia	-		Ali et al. (2017)
	*Dysphania ambrosioides* (L.) Mosyakin & Clemants	Paico	(sho)	Peru#	Uta	^p^ *Lm* IC_50_>100 μg/ml	Kvist et al. (2006)
	(2 quotes)	Paico	(le)	Ecuador	Cutaneous leishmaniasis	-	Weigel et al. (1994)
	*Iresine diffusa* Humb. & Bonpl. ex Willd.	-	(le, to)	Ecuador#	-		Gachet et al. (2010)
Amaryllidaceae (4 quotes and 4 species)	*Allium cepa* L.	Cebolla Paitena	(le/sta)	Ecuador	Cutaneous leishmaniasis	-	Weigel et al. (1994)
	*Allium sativum* L.	Ajo	(cl)				
	*Crinum* *sp.*	-	(ro, to)	Ecuador#	-		Gachet et al. (2010)
	*Scadoxus multiflorus* (Martyn) Raf.	Dem Astefi	po (ro, to)	Ethiopia	‘Gurtb’ leishmaniasis		Teklehaymanot, (2009)
Anacardiaceae (5 quotes and 3 species)	*Mangifera indica* L. (2 quotes)	Mango	(co)	Peru#	Uta	^p^ *Lm* IC_50_>100 μg/ml	Kvist et al. (2006)
		Mã	po (ba, to)	French Guiana (Wayãpi)	Leishmaniasis	-	Odonne et al. (2011a)
	*Spondias mombin* L. (2 quotes)	Ubos	dec (ba, to/or)	Peru (Chayahuita)	Uta	^*p*^ *La*> 100 μg/ml	Estevez et al. (2007)
						^a^ *La*> 100 μg/ml	
			(co)	Peru#	Uta	^p^ *Lm -* NA	Kvist et al. (2006)
	*Spondias purpurea* L.	-	(ba, to)	Ecuador#	-	-	Gachet et al. (2010)
Annonaceae (2 quotes and 2 species)	*Annona ambotay* Aubl.	Iwitay	po (ba, to)	French Guiana (Wayãpi)	Leishmaniasis	-	Odonne et al. (2011a)
	*Cremastosperma longicuspe* R.E.Fr.	Maya Sohuit	Pow-po (ba, to)	Peru (Chayahuita)	-	^a^ *La*> 100 μg/ml	Odonne et al. (2009)
Apocynaceae (9 quotes and 7 species)	*Aspidosperma excelsum* Benth.	Remo Caspi (De Baja)	(co)	Peru#	Uta	^p^ *Lm –* NA	Kvist et al. (2006)
	*Aspidosperma rigidum* Rusby	Gabetillo	po (st/ba, to)	Bolivia#	Cutaneous leishmaniasis	-	Hajdu and Hohmann, (2012)
	*Himatanthus articulatus* (Vahl) Woodson	Compuhuan	po (ba, to)	Peru (Chayahuita)	-		Odonne et al. (2009)
	*Tabernaemontana flavicans* Roem. & Schult.	Shinanpi	Pow-po (ba, to)				
	*Tabernaemontana sananho* Ruiz & Pav. (3 quotes)	Shinambik	Fresh-po (ro, to)		Uta	^*p*^ *La* = 9 μg/ml	Estevez et al. (2007)
						^a^ *La* = 58 μg/ml	
		-	(ba, to)	Ecuador#	-	-	Gachet et al. (2010)
		Shinanp	pow-po (ba, to)	Peru (Chayahuita)			Odonne et al. (2009)
	*Tabernaemontana siphilitica* (L.f.) Leeuwenb	Radie Capiaye	po (lt, to)	French Guiana	Leishmaniasis		Odonne et al. (2011a)
	*Tabernaemontana* *sp*.	Lobo sanango	(Ro)	Peru#	Uta	^p^ *Lm*	Kvist et al. (2006)
						IC_50_ = 15 μg/ml	
Araceae (23 quotes and 16 species)	*Anthurium muyunense* Croat.	Shimpanantë	dec (to)	Peru (Chayahuita)	-	-	Odonne et al. (2009)
	*Anthurium* *sp*.	-	(le, to)	Ecuador#			Gachet et al. (2010)
	*Caladium bicolor* (Aiton) Vent.	Ahtata’Ta	po, (ro, to)	Peru (Chayahuita)	Uta	^*p*^ *La* - ΝΑ	Estevez et al. (2007)
						^a^ *La* IC_50_>100 μg/ml	
	*Caladium picturatum* K.Koch & C.D.Bouché.	Io Ata’	po, (tu, to)		Ta’Ta’	^a^ *La* IC_50_>100 μg/ml	Odonne et al. (2009)
	*Colocasia esculenta* (L.) Schott.	-	(le, to)	Ecuador#	-	-	Gachet et al. (2010)
	*Dieffenbachia seguine* (Jacq.) Schott	Patiquina, Hoja Blanca	Inf-po (st, to)	Peru	Uta		Vásquez-Ocmín et al. (2018)
	*Dieffenbachia williamsii* Croat (2 quotes)	Corech	dec-po (wp/le, to)	Peru (Yanesha)	Uta De Agua, Mareñets	^a^ *La* IC_50_>100 μg/ml	Valadeau et al. (2009)
			dec-po (wp, to)		Cutaneous Leishmaniasis, Wound that Do not heal	-	
	*Dieffenbachia* sp*.* (4 quotes)	-	(le, to)	Ecuador#	-		Gachet et al. (2010)
		Mata Boro	po (st/ba, to)	Bolivia#	Cutaneous leishmaniasis		Hajdu and Hohmann, (2012)
		Patiquina	(le)	Peru#	Uta	^p^ *Lm* IC_50_>100 μg/ml	Kvist et al. (2006)
		Shimpan	dec-po (st, to)	Peru (Chayahuita)	-	-	Odonne et al. (2009)
	*Dracontium spruceanum* (Schott) G.H.Zhu.	Jergón Sacha, Hierba Del Jergón,	pow-po (tu, to)	Peru	Uta		Vásquez-Ocmín et al. (2018)
		Fer De Lance					
	*Philodendron surinamense* (Miq.) Engl.	Huambe	*“Is Drunk In Small Quantities Three Times Daily”* dec (ro, or)	Peru (Chayahuita)	Uta	^p^ *La* IC_50_>100 μg/ml	Estevez et al. (2007)
						^a^ *La* IC_50_>100 μg/ml	
	*Philodendron* sp*.* (3 quotes)	-	(le, to)	Ecuador#	-	-	Gachet et al. (2010)
			(le, to)				
			(le, to)				
	*Pistia stratiotes* L.	Puto puto	(le)	Peru#	Uta	^p^ *Lm -* NA	Kvist et al. (2006)
	*Rhodospatha* *sp*.	-	(le, to)	Ecuador#	-	-	Gachet et al. (2010)
	*Stenospermation* sp*.* (2 quotes)		(le, to)				
			(le, to)				
	*Thaumatophyllum solimoesense *(A.C.Sm.) Sakur., Calazans & Mayo.	Huambe	*“Is Drunk In Small Quantities Three Times Daily”* dec (ro, or)	Peru (Chayahuita)		^p^ *La* IC_50_>100 μg/ml	Estevez et al. (2007)
						^a^ *La* IC_50_>100 μg/ml	
	*Xanthosoma* *sp*.	-	(le, to)	Ecuador#	-	-	Gachet et al. (2010)
Arecaceae (1 quote and 1 species)	*Euterpe oleracea* Mart.	Wasey	fresh-po (am/ro, to)	French Guiana (Wayãpi)	Leishmaniasis	-	Odonne et al. (2011a)
Aspleniaceae – Pteridophyta (1 quote and 1 species)	*Thelypteris* *sp*.	-	(le, to)	Ecuador#	-	-	Gachet et al. (2010)
Asteraceae (22 quotes and 20 species)	*Achillea arabica* Kotschy.	Aldefera	fresh-po (ap, to)	Saudi Arabia	Leishmania	-	Ali et al. (2017)
	*Acmella brachyglossa* Cass.	-	(le, to)	Ecuador#	-		Gachet et al. (2010)
	*Adenostemma brasilianum* Cass.						
	*Ageratum conyzoides* L.						
	*Baccharis sagittalis* (less.) DC.	Charara	(wp/le, to)	Bolivia (Kechua)	*Espundia* (cutaneous and mucocutaneous leishmaniasis)	^p^ *La -* NA	Fournet et al. (1994)
						^*p*^ *Lb -* NA	
						^p^ *Ld -*NA	
	*Bidens pilosa* L.	-	(se, to)	Ecuador#	-	-	Gachet et al. (2010)
	*Clibadium cf. microcephalum* S.F.Blake.		(le/st, to)				
	*Erigeron* *sp*.						
	*Elephantopus mollis* Kunth.		(wp, to)				
	*Erigeron bonariensis* L.						
	*Eupatorium* *sp*.						
	*Matricaria chamomilla* L.	Manzanilla	(fL)	Ecuador	Cutaneous leishmaniasis		Weigel et al. (1994)
	*Mikania* *sp*.	-	(le, to)	Ecuador#	-		Gachet et al. (2010)
	*Munnozia hastifolia* (Poepp.) H. Rob. & Brettell.	Huallapnarren	fresh-po (le, to)	Peru (Yanesha)	Uta De Agua, Mareñets	^a^ *La* IC_50_ = 14.1 μg/ml	Valadeau et al. (2009)
	(2 quotes)		fresh-po (lt, to)		Leishmaniasis	-	Valadeau et al. (2010)
	*Piptocoma discolor* (Kunth) Pruski.	-	(le, to)	Ecuador#	-		Gachet et al. (2010)
	*Porophyllum ruderale* (Jacq.) Cass.	Ebus'A Ina,	pow-po, (le, to)	Bolivia (Takana indians)	Leishmaniasis	^p^ *La*IC_50_ > 100μg/mL^p^ *Lb*IC_50_ > 100 μg/ml	Arévalo-Lopéz et al. (2018)
		Chadhi Ina					
	*Pseudelephantopus spicatus* (Juss. ex Aubl.) C.F.Baker.	Huapato, Pato, Cahuario Pacatro		Peru (Chayahuita)	Ta’Ta’	^a^ *La*	Odonne et al. (2009)
	(2 quotes)	Wapatu, Cawariu Pacaturu, Patu				IC_50_ = 27.3 μg/ml	(Odonne et al., 2011b)
	*Taraxacum campylodes* G.E.Haglund	-	(le, to)	Ecuador#	-	-	Gachet et al. (2010)
	*Tessaria integrifolia* Ruiz	Cawuara	fresh-po (le, to)	Bolivia (Takana indians)	Leishmaniasis	^p^ *La* IC_50_ = 54.2μg/mL^p^ *Lae*IC_50_ = 48μg/mL^p^ *Lb* IC_50_ = 31.6μg/mL^p^ *Lla* IC_50_ = 34.8 μg/ml	Arévalo-Lopéz et al. (2018)
	& Pav.						
	*Vernonanthura patens* (Kunth) H.Rob.	-	(le/st, to)	Ecuador#	-	-	Gachet et al. (2010)
Begoniaceae (1 quote and 1 species)	*Begonia* *sp*.	-	(st, to)	Ecuador#	-	-	Gachet et al. (2010)
Bignoniaceae (13 quotes and 10 species)	*Callichlamys latifolia* (rich.) K. Schum.	Kalasapau Poã Ipo Pilã	fresh-po (ba, to)	French Guiana (Wayãpi)	Leishmaniasis	-	Odonne et al. (2011a)
	*Crescentia cujete* L. (2 quotes)	Kwi’I	po (ba, to)				
		-	(le, or)	Ecuador#	-		Gachet et al. (2010)
	*Fridericia nigrescens* (Sandwith) L.G.Lohmann.	Kalasapau Poã Ipo	fresh-po (ba, to)	French Guiana (Wayãpi)	Leishmaniasis		Odonne et al. (2011a)
	*Handroanthus impetiginosus* (mart. ex DC.) Mattos.	Tahuari	“Boil 200 G of the bark In 1 L *Of Water. Wash The Affected Area And Apply As A Compress Until Cicatrization Of The Ulcers”* dec-po (ba, to)	Peru	Uta		Vásquez-Ocmín et al. (2018)
	*Jacaranda copaia* (Aubl.) D.Don.	Charapachpan	dec-po (le, to)	Peru (Yanesha)	Uta De Agua, Mareñets	^a^ *La* IC_50_ = 16.5 μg/ml	Valadeau et al. (2009)
	(2 quotes)				Leishmaniasis	-	Valadeau et al. (2010)
	*Jacaranda cuspidifolia* mart.	Arabisco	(le, to)	Bolivia (Mozetenes, tacanas or Chimanes indians, and other ethnic groups)	*Espundia* (cutaneous and mucocutaneous leishmaniasis)	^p^ *La;* ^*p*^ *Lb;* ^p^ *Ld*	Fournet et al. (1994)
	*Jacaranda glabra* (DC) Bureau & K. Schum.	Chepere Qui	dec-po (ba/le/fr, to)	Bolivia (Takana indians)	Leishmaniasis	^p^ *La* IC_50_ = 29.8 μg/ml	Arévalo-Lopéz et al. (2018)
						^p^ *Lae*	
						IC_50_ = 45.4 μg/ml	
						^p^ *Lb* IC_50_ = 17.4 μg/ml	
						^p^ *Lla* IC_50_ = 27.5 μg/ml	
	(2 quotes)	-	(le, to)	Ecuador#	-	-	Gachet et al. (2010)
	*Mansoa alliacea* (Lam.) A.H. Gentry.	Ananan	pow-po (le, to)	Peru (Chayahuita)	Ta’Ta’		Odonne et al. (2009)
	*Mansoa standleyi* (Steyerm.) A.H. Gentry.	Ajo sacha (macho)	(ro)	Peru#	Uta	^p^ *Lm*	Kvist et al. (2006)
						IC_50_ = 18 μg/ml	
	*Mansoa* *sp*.	Ajo Silvestre, De Monte, Sacha, Kofan: Cumpanafema, Palobrea	-	Colombia (Kofan)	Cutaneous leishmaniasis	-	Gutiérrez et al. (2014)
Bixaceae (2 quotes and 1 species)	*Bixa orellana* L. (2 quotes)	Uluku	fresh-po (se, to)	French Guiana (Wayãpi)	Leishmaniasis	-	Odonne et al. (2011a)
		Achiote	(le)	Ecuador	Cutaneous leishmaniasis		Weigel et al. (1994)
Bromeliaceae (1 quote and 1 species)	*Billbergia decora* Poepp. & Endl.	Nara Shimpanantë	fresh-po (st, to)	Peru (Chayahuita)	-	-	Odonne et al. (2009)
Burseraceae (1 quote and 1 species)	*Commiphora gileadensis* (L.) C.Chr.	Al-Bisham	fresh-po (or, to)	Saudi Arabia	Leishmaniasis	-	Ali et al. (2017)
Cactaceae (1 quote and 1 species)	*Cereus hexagonus* (L.) Mill.	Kau Kau	fresh-po (ba, to)	French Guiana (Wayãpi)	Leishmaniasis	-	Odonne et al. (2011a)
Cannabaceae (2 quotes and 2 species)	*Trema integerrima* (Beurl.) Standl.	-	(le/st, to)	Ecuador#	-	-	Gachet et al. (2010)
	*Trema micrantha* (L.) Blume.	Surrumbo, Veraquillo	-	Colombia (Kofan)	Cutaneous leishmaniasis		Gutiérrez et al. (2014)
Caricaceae (4 quotes and 1 species)	*Carica papaya* L. (4 quotes)	-	(ba/le, to)	Ecuador#	-	-	Gachet et al. (2010)
		Papaye (Bapaju) (Mã˜ U)	fresh-po (lt, to)	French Guiana (Wayãpi, Teko)	Leishmaniasis	-	Odonne et al. (2011a)
		Papaypan		Peru (Yanesha)	Uta De Agua, Mareñets	^a^ *La* IC_50_ = 11.2 μg/ml	Valadeau et al. (2009)
		Papaya			Leishmaniasis	-	Valadeau et al. (2010)
Celastraceae (4 quotes and 3 species)	*Maytenus macrocarpa* (Ruiz & Pav.) Briq.	Shoshohuasha	pow-po (ba, to)	Peru (Chayahuita)	Ta’Ta’	-	Odonne et al. (2009)
	(2 quotes)	Chuchuhuasi, Chuchuhuasha	dec-po (ba, to)	Peru	Uta		Vásquez-Ocmín et al. (2018)
	*Maytenus* *sp*.	Chuchuhuasi (Del Bajo)	(co)	Peru#	Uta	^p^ *Lm*	Kvist et al. (2006)
						IC_50_ = 10–20 μg/ml	
	*Salacia juruana* Loes.	Shoshohuasha Nonin	pow-po (ba, to)	Peru (Chayahuita)	Ta’Ta’	^a^ *La*	Odonne et al. (2009)
						IC_50_ = 41 μg/ml	
Combretaceae (1 quote and 1 species)	*cf Combretum* *sp*.	Ipoyu	fresh-po (sa, to)	French Guiana (Teko)	Leishmaniasis	-	Odonne et al. (2011a)
Commelinaceae (2 quotes and 2 species)	*Dichorisandra hexandra* (Aubl.) C.B.Clarke.	-	(le/st/wp, to)	Ecuador#	-	-	Gachet et al. (2010)
	*Dichorisandra* *sp*.		(st, to)				
Convolvulaceae (1 quote and 1 species)	*Ipomoea* *sp*.	-	(le, to)	Ecuador#	-	-	Gachet et al. (2010)
Costaceae (1 quote and 1 species)	*Costus* *sp*.	-		Ecuador#	-	-	Gachet et al. (2010)
Crassulaceae (2 quotes and 2 species)	*Kalanchoe gastonis-bonnieri* Raym.- Hamet & H. Perrier.	-	(le, to)	Ecuador#	-	-	Gachet et al. (2010)
	*Kalanchoe pinnata* (Lam.) pers		(le, or/to)				
Cucurbitaceae (4 quotes and 3 species)	*Cayaponia* *sp.*	-	(le, to)	Ecuador#	-	-	Gachet et al. (2010)
	*Gurania lobata* (L.) Pruski.						
	*Gurania* sp*.* (2 quotes)						
		Hoja Ancha (Kofan, Putumayoc Colombia)	-	Colombia (Kofan)	Cutaneous leishmaniasis		Gutiérrez et al. (2014)
Cyclanthaceae (1 quote and 1 species)	*Cyclanthus* *sp*.	-	-	Colombia (Kofan)	Cutaneous leishmaniasis	-	Gutiérrez et al. (2014)
Dilleniaceae (1 quote and 1 species)	*Doliocarpus* *sp*.	-	(le, to)	Ecuador#	-	-	Gachet et al. (2010)
Equisetaceae (1 quote and 1 species)	*Equisetum bogotense* Kunth.	-	(st, to)	Ecuador#	-	-	Gachet et al. (2010)
Euphorbiaceae (21 quotes and 15 species)	*Acalypha alopecuroidea* Jacq.	-	(wp, to)	Ecuador#	-	-	Gachet et al. (2010)
	*Acalypha diversifolia* Jacq.	Sanquemula	-	Colombia (Kofan)	Cutaneous leishmaniasis		Gutiérrez et al. (2014)
	*Acalypha macrostachya* Jacq. (2 quotes)	Mareñtsopar	fresh-po (lt, to)	Peru (Yanesha)	Uta De Agua, Mareñets	^a^ *La* IC_50_ = 32.9 μg/ml	Valadeau et al. (2009)
					Leishmaniasis	-	Valadeau et al. (2010)
	*Croton draconoides* Müll.Arg.	Sangre de Grado	fresh-po, (re, to)	Bolivia#	Cutaneous leishmaniasis		Hajdu and Hohmann (2012)
	*Croton lechleri* Müll.Arg. (2 quotes)	-	(ex, to)	Ecuador#	-		Gachet et al. (2010)
		Sangre de Drago	(re)	Peru#	Uta	^p^ *Lm* IC_50_>100 μg/ml	Kvist et al. (2006)
	*Croton* *sp*.		-	Colombia (Kofan)	Cutaneous leishmaniasis	-	Gutiérrez et al. (2014)
	*Euphorbia ampliphylla* Pax.	Adami	resh-po (sa, to)	Ethiopia (Oromo)			Suleman and Alemu (2012)
	*Euphorbia heterophylla* L. (2 quotes)	T Ate’Ñeñt	fresh-po (st/le, to)	Peru (Yanesha)	Uta De Agua, Mareñets	^a^ *La* IC_50_ = 25.6 μg/ml	Valadeau et al. (2009)
			fresh-po, (lt, to)		Leishmaniasis	-	Valadeau et al. (2010)
	*Euphorbia* *sp*.	-	(le/st, to)	Ecuador#	-		Gachet et al. (2010)
	*Hura crepitans* L. (3 quotes)	Catahua	(re)	Peru#	Uta	^p^ *Lm* IC_50_>100 μg/ml	Kvist et al. (2006)
		Nëquëra	pow-po (ba, to)	Peru (Chayahuita)	Ta’Ta’	-	Odonne et al. (2009)
		Soliman	(lt, to)	Bolivia (Chimane indians)	*Espundia* (cutaneous and mucocutaneous leishmaniasis)	^p^ *La*; ^*p*^ *Lb*; ^p^ *Ld*	Fournet et al. (1994)
	*Jatropha curcas* L. (2 quotes)	Shanëquëra	fresh-po (lt, to)	Peru (Chayahuita)	Ta’Ta’	-	Odonne et al. (2009)
		Kalasapau Poã	fresh-po (ba/fr/ro, to)	French Guiana (Wayãpi)	Leishmaniasis		Odonne et al. (2011a)
	*Manihot esculenta* Crantz.	-	(le, to)	Ecuador#	-		Gachet et al. (2010)
	*Maprounea guianensis* Aubl.	Ka’Asili	po (le, to)	French Guiana (Wayãpi)	Leishmaniasis		Odonne et al. (2011a)
	*Sapium ciliatum* Hemsl.	Melekene Sili	fresh-po (lt/ba, to)				
	*Sapium marmieri* Huber.	Tocaï	fresh-po (lt, to)	Peru (Chayahuita)	-		Odonne et al. (2009)
Fabaceae (27 quotes and 23 species)	*Acacia* *sp*.	Wikamallki	(le, to)	Bolivia (Kechua)	*Espundia* (cutaneous and mucocutaneous leishmaniasis)	^p^ *La*; ^*p*^ *Lb*; ^p^ *Ld*	Fournet et al. (1994)
	*Bauhinia tarapotensis* Benth.	-	(le/st, or/to)	Ecuador#	-	-	Gachet et al. (2010)
	*Cajanus cajan* (L.) Huth.		(Ba, to)				
	*Campsiandra angustifolia* Spruce ex Benth.	Huacapurana	(co)	Peru#	Uta	^p^ *Lm* IC_50_>100 μg/ml	Kvist et al. (2006)
	*Cassia* *sp*.	-	(le/st, to)	Ecuador#	-	-	Gachet et al. (2010)
	*Copaifera officinalis* L.	Bálsamo, copaiba	-	Colombia (Kofan)	Cutaneous leishmaniasis		Gutiérrez et al. (2014)
	*Copaifera paupera* (Herzog) Dwyer.	Nampihuora	fresh-po (sa, to)	Peru (Chayahuita)	Uta	^p^ *La* IC_50_>100 μg/ml	Estevez et al. (2007)
						^a^ *La* IC_50_>100 μg/ml	
		Copaiba	(re)	Peru#	Uta	^p^ *Lm* - NA	Kvist et al. (2006)
		Nanpihuara	fresh (re, or/to)	Peru (Chayahuita)	Ta’Ta’	-	Odonne et al. (2009)
		Copaiba	*“Take Five Drops Of Oil (Exsudate) Diluted In A Tablespoon Of Warm Water, On An Empty Stomach, For Seven Days”* fresh (sa, or)	Peru	Uta		Vásquez-Ocmín et al. (2018)
	*Deguelia chrysophylla* (Kleinhoonte) R.A.Camargo & A.M.G.Azevedo.	Imeku	po	French Guiana (Wayãpi)			Odonne et al. (2011a)
	*Desmodium axillare* (Sw.) DC.	Së’Ë	pow-po (le, to)	Peru (Chayahuita)	Ta’Ta’	^a^ *La*	Odonne et al. (2009)
						IC_50_ = 17 μg/ml	
	*Erythrina* *sp*.	Flor De Mayo	(st, to)	Bolivia	*Espundia* (cutaneous and mucocutaneous leishmaniasis)	^p^ *La*; ^*p*^ *Lb*; ^p^ *Ld*	Fournet et al. (1994)
	*Grona adscendens* (Sw.) H.Ohashi & Ohashi.	-	(le/st/wp/fr, or/to)	Ecuador#	-	-	Gachet et al. (2010)
	*Hydrochorea corymbosa* (Rich.) Barneby & J.W.Grimes.	Kalai Pei	po (ba, to)	French Guiana (Teko)	Leishmaniasis		Odonne et al. (2011a)
	*Inga bourgonii* (Aubl.) DC.	Inga Sisi, Bougouni		French Guiana (Wayãpi, Teko)			
	*Inga edulis* Mart. (2 quotes)	Inga Wasa		French Guiana (Wayãpi)			
		-	(le, to)	Ecuador#	-		Gachet et al. (2010)
	*Inga oerstediana* Benth.		(ba/le, to)				
	*Inga* *sp*.	Inga U	po (ba, to)	French Guiana (Mixed Wayãpi/Teko)	Leishmaniasis		Odonne et al. (2011a)
	*Lonchocarpus seorsus* (J.F. Macbr.) M. Sousa ex D.A. Neill, Klitg. & G.P. Lewis.	-	(ba, to)	Ecuador#	-		Gachet et al. (2010)
	*Lupinus tauris* Benth.	Tauri	(le)	Ecuador	Cutaneous leishmaniasis		Weigel et al. (1994)
	*Mucuna* *sp*.	-	(ba, or/to)	Ecuador#	-		Gachet et al. (2010)
	*Myroxylon balsamum* (L.) Harms.		(ba, to)				
	*Phaseolus* *sp*.		(le/st, to)				
	*Piptadenia* *sp*.		(le, to)				
	*Senna reticulata* (Willd.) H.S.Irwin & Barneby.	Pole	Inf-po (le)	french Guiana (Wayãpi)	Leishmaniasis		Odonne et al. (2011a)
Gentianaceae (4 quotes and 2 species)	*Coutoubea ramosa* Aubl.	Mamanwã Puã	fresh-po (le, to)	French Guiana (Teko)	Leishmaniasis	-	Odonne et al. (2011a)
	*Helia alata* (Aubl.) Kuntze. (3 quotes)	Puepa’ ∼Tpan	fresh-po (le, to)	Peru (Yanesha)	Leishmaniasis		Valadeau et al. (2010)
		Campanita	dec-po, (le, or)	Peru	Uta		Vásquez-Ocmín et al. (2018))
		Puepa’T˜Pan	fresh-po (le, to)	Peru (Yanesha)	Uta De Agua, Mareñets	^a^ *La* IC_50_ = 37.4 μg/ml	Valadeau et al. (2009)
Gesneriaceae (2 quotes and 2 species)	*Drymonia turrialvae* Hanst.	-	(le, to)	Ecuador#	-	-	Gachet et al. (2010)
	*Drymonia* *sp*.		(wp, to)				
Haemodoraceae (1 quote and 1 species)	*Xiphidium caeruleum* Aubl.	-	(le, to)	Ecuador#	-	-	Gachet et al. (2010)
Heliconiaceae (1 quote and 1 species)	*Heliconia stricta* Huber.	Tanan Tancomë	fresh-po (ro, to)	Peru (Chayahuita)	-	-	Odonne et al. (2009)
Hypericaceae (2 quotes and 1 species)	*Vismia* sp*.* (2 quotes)	Mareñtsorech	fresh-po (lt, to)	Peru (Yanesha)	Leishmaniasis	-	Valadeau et al. (2010)
			fresh-po, (st, to)		Uta De Agua, Mareñets	^a^ *La* IC_50_ = 54.3 μg/ml	Valadeau et al. (2009)
Iridaceae (1 quote and 1 species)	*Eleutherine bulbosa* (Mill.) Urb.	Wasey Laãnga	fresh-po, (bu, to)	French Guiana (Wayãpi)	Leishmaniasis	-	Odonne et al. (2011a)
Lamiaceae (9 quotes and 7 species)	*Cantinoa mutabilis* (rich.) Harley & J.F.B.Pastore.	-	(le/wp, to)	Ecuador#	-	-	Gachet et al. (2010)
	(2 quotes)	Tapacha Ina	pow-po (le/ro, to)	Bolivia (Takana indians)	Leishmaniasis	^p^ *La* IC_50_ = 29.7 μg/ml	Arévalo-Lopéz et al. (2018)
						^p^ *Lb* IC_50_ = 9.8 μg/ml	
	*Hyptis capitata* Jacq.	-	(le, to)	Ecuador#	-	-	Gachet et al. (2010)
	*Hyptis lacustris* A.St.-Hil. ex Benth. (2 quotes)	Ollamepan	fresh-po (st/le, to)	Peru (Yanesha)	Uta De Agua, Mareñets	^a^ *La*	Valadeau et al. (2009)
						IC_50_ = 10 μg/ml	
			fresh-po (le, to)		Leishmaniasis	-	Valadeau et al. (2010)
	*Mesosphaerum pectinatum* (L.) Kuntze.	-	(le/wp, to)	Ecuador#	-	-	Gachet et al. (2010)
	*Minthostachys* *sp*.		(le/fr, to)				
	*Ocimum campechianum* Mill.		(le, to)				
	*Salvia* *sp*.						
Lecythidaceae (4 quotes and 3 species	*Couroupita guianensis* Aubl. (2 quotes)	-	(Fr, to)	Ecuador#	-	-	Gachet et al. (2010)
		Aya huma	(co)	Peru#	Uta	^p^ *Lm* IC_50_>100 μg/ml	Kvist et al. (2006)
	*Grias neuberthii* J.F.Macbr.	-	(se, to)	Ecuador#	-	-	Gachet et al. (2010)
	*Grias peruviana* Miers.	Anpi	fresh-po (fr/ba, to)	Peru (Chayahuita)	Ta’Ta’, Huayani		Odonne et al. (2009)
Loasaceae (1 quote and 1 species)	*Klaprothia fasciculata* (C. Presl) Poston.	-	(le/st, to)	Ecuador#	-	-	Gachet et al. (2010)
Loganiaceae (1 quote and 1 species)	*Strychnos* *sp*.	-	(se, to)	Ecuador#	-	-	Gachet et al. (2010)
Loranthaceae (1 quote and 1 species)	*Struthanthus* *sp*.	-	(wp, to)	Ecuador#	-	-	Gachet et al. (2010)
Malpighiaceae (1 quote and 1 species)	*Banisteriopsis caapi* (Spruce ex Griseb.) Morton.	-	(le/st, to)	Ecuador#	-	-	Gachet et al. (2010)
Malvaceae (11 quotes and 11 species)	*Abutilon* *sp*.	-	(le, to)	Ecuador#	-	-	Gachet et al. (2010)
	*Ceiba pentandra* (L.) Gaertn.	Kumaka	fresh-po (ba, to)	French Guiana (Wayãpi)	Leishmaniasis	-	Odonne et al. (2011a)
	*Gossypium barbadense* L.	Coton Violet	fresh-po (fl/le, to)	French Guiana (Brazilian and mixed Wayãpi/Teko)			
	*Gossypium* *sp*.	Jirbi (O) Tit (A)	*“The Seed Is Powdered And Pasted With Butter”* pow-po (se, to)	Ethiopia (Oromo)	Cutaneous leishmaniasis		Suleman and Alemu (2012)
	*Hibiscus rosa-sinensis* L.	-	(le, to)	Ecuador#	-		Gachet et al. (2010)
	*Hibiscus sabdariffa* L.		po (le/st, to)	French Guiana (Wayãpi)	Leishmaniasis		Odonne et al. (2011a)
	*Hibiscus* *sp*.		(le, to)	Ecuador#	-		Gachet et al. (2010)
	*Matisia cordata* Bonpl.		(le, to)	Ecuador#			Gachet et al. (2010)
	*Pavonia fruticosa* (Mill.) Fawc. and Rendle.	Sëncopi Së’Ë	fresh-po (le, to)	Peru (Chayahuita)			Odonne et al. (2009)
	*Sida rhombifolia* L.	Escobilla	(le)	Ecuador	Cutaneous leishmaniasis		Weigel et al. (1994)
	*Theobroma cacao* L.	-	(se, to)	Ecuador#	-		Gachet et al. (2010)
Marantaceae (2 quotes and 2 species)	*Calathea* *sp*.	Tumbaje (Kofan, Putumayoc Colombia)	-	Colombia (Kofan)	Cutaneous leishmaniasis	-	Gutiérrez et al. (2014)
	*Ischnosiphon* *sp*.	-	(le, to)	Ecuador#	-		Gachet et al. (2010)
Melastomataceae (6 quotes and 5 species)	*Adelobotrys* *sp*.	-	(wp, to)	Ecuador#	-	-	Gachet et al. (2010)
	*Antherotoma senegambiensis* (Guill. & Perr.) Jacq.-Fél.		(le, na)	Ethiopia (Meinit)	Cutaneous leishmaniasis		Giday et al. (2009)
	*Clidemia allardii* Wurdack.		(le, to)	Ecuador#	-		Gachet et al. (2010)
	*Miconia* sp*.* (2 quotes)						
	*Tococa guianensis* Aubl.						
Meliaceae (5 quotes and 2 species)	*Carapa guianensis* Aubl.	Yani	fresh-po (ba/se, to)	French Guiana (Wayãpi)	Leishmaniasis	-	Odonne et al. (2011a)
	*Cedrela odorata* L. (4 quotes)	Cedro	dec-bath (ba, to)	Peru	Uta		Vásquez-Ocmín et al. (2018)
		-	(ba/le, or/to)	Ecuador#	-		Gachet et al. (2010)
		Cedro	(co)	Peru#	Uta	^p^ *Lm*	Kvist et al. (2006)
						IC_50_ = 60 μg/ml	
		Nonara	pow-po (ba, to)	Peru (Chayahuita)	Ta’Ta’	-	Odonne et al. (2009)
Menispermaceae (2 quotes and 1 species)	*Curarea tecunarum* Barneby and Krukoff. (2 quotes)	Abuta	(st)	Peru#	Uta	^p^ *Lm*	Kvist et al. (2006)
						IC_50_ > 100 μg/ml	
		Capari Nonirintë	pow-po (ba, to)	Peru (Chayahuita)	Ta’Ta’	-	Odonne et al. (2009)
Meteoriaceae (1 quote and 1 species)	*Meteoridium* *sp*.	-	(wp, to)	Ecuador#	-	-	Gachet et al. (2010)
Metteniusaceae (1 quote and 1 species)	*Poraqueiba sericea* Tul.	Umarí	(co)	Peru#	Uta	^p^ *Lm*	Kvist et al. (2006)
						IC_50_>100 μg/ml	
Moraceae (7 quotes and 6 species)	*Artocarpus altilis* (Parkinson) Fosberg.	-	(le, to)	Ecuador#	-	-	Gachet et al. (2010)
	*Castilla elastica* Cerv.	Caucho Negro		Colombia (Afro-Colombian and indigenous groups)		^p^ *La -*NA	Weniger et al. (2001)
						^p^ *Lb -*NA	
						^p^ *Li -* NA	
						^*a*^ *Lp -*NA	
	*Dorstenia foetida* Schweinf	Om -Lakef	bath (to)	Saudi Arabia		-	Ali et al. (2017)
	*Ficus dendrocida* Kunth.	Matapalo	(as)	Ecuador	Cutaneous leishmaniasis		Weigel et al. (1994)
	*Ficus insipida* Willd. (2 quotes)	Ojé	(re)	Peru#	Uta	^p^ *Lm*	Kvist et al. (2006)
						IC_50_ > 100 μg/ml	
		Ojé, Doctor Ojé	fresh-po (lt, to)	Peru	Uta	-	Vásquez-Ocmín et al. (2018)
	*Ficus* *sp*.	Matapalo	(lt, to)	Bolivia	*Espundia* (cutaneous and mucocutaneous leishmaniasis)	^p^ *La*; ^*p*^ *Lb*; ^p^ *Ld*	Fournet et al. (1994)
Musaceae (5 quotes and 2 species)	*Musa acuminata* Colla.	-	(le, to)	Ecuador#	-	-	Gachet et al. (2010)
	*Musa × paradisiaca* L. (4 quotes)	Pako	po (to)	French Guiana (Wayãpi)	Leishmaniasis		Odonne et al. (2011a)
		-	(fr, to)	Ecuador#	-		Gachet et al. (2010)
		Plátano	(sa/fr)	Ecuador	Cutaneous leishmaniasis		Weigel et al. (1994)
		Pantapi	pow-po (fr, to)	Peru (Chayahuita)	Ta’Ta’		Odonne et al. (2009)
Myristicaceae (3 quotes and 3 species)	*Otoba novogranatensis* Moldenke.	Otobo	(re, to)	Colombia (Afro-Colombian and indigenous groups)	-	^p^ *La +*	Weniger et al. (2001)
	*Otoba parvifolia* (Markgr.) A.H.Gentry.		fresh-po (re, to)			^p^ *Lb +* ^p^ *Li +* ^*a*^ *Lp +*	
	*Virola surinamensis* (Rol. ex Rottb.) Warb.	Cumala Colorada	*“Boil 5 G Of The Bark In One Liter Of Water. Drink One Cup Every Morning For Three Days”* dec (ba, or)	Peru	Uta	-	Vásquez-Ocmín et al. (2018)
Myrtaceae (4 quotes and 3 species)	*Myrtus communis* L.	Al-A’S	fresh-po (le, to)	Saudi Arabia	-	-	Ali et al. (2017)
	*Psidium acutangulum* DC.	Alali (Goyave Saut)	fresh-po (ba, to)	French Guiana (Wayãpi)	Leishmaniasis		Odonne et al. (2011a)
	*Psidium guajava* L. (2 quotes)	-	(ba/le, to)	Ecuador#	-		Gachet et al. (2010)
		Guayaba	-	Ecuador	Cutaneous leishmaniasis		Weigel et al. (1994)
Olacaceae (2 quotes and 1 species)	*Minquartia guianensis* Aubl. (2 quotes)	Huacapú	(co)	Peru#	Uta	^p^ *Lm*	Kvist et al. (2006)
						IC_50_ < 10 μg/ml	
		-	(ba/le, to)	Ecuador#	-	-	Gachet et al. (2010)
Oleaceae (1 quote and 1 species)	*Olea europaea* L.	Al-aotem	inf-po (st, to)	Saudi Arabia	-	-	Ali et al. (2017)
Onagraceae (1 quote and 1 species)	*Ludwigia* *sp*.	-	(le/st, to)	Ecuador#	-	-	Gachet et al. (2010)
Oxalidaceae (1 quote and 1 species)	*Oxalis* *sp*.	‘Sebastian’	(le, to)	Bolivia	*Espundia* (cutaneous and mucocutaneous leishmaniasis)	^p^ *La*; ^*p*^ *Lb*; ^p^ *Ld*	Fournet et al. (1994)
Papaveraceae (1 quote and 1 species)	*Bocconia integrifolia* Bonpl.	Palo Amarillo/Amakari	(le/lt/st, to)	Bolivia (Kechua)	*Espundia* (cutaneous and mucocutaneous leishmaniasis)	^p^ *La*; ^*p*^ *Lb*; ^p^ *Ld*	Fournet et al. (1994)
Peraceae (2 quotes and 1 species)	*Pera benensis* Rusby. (2 quotes)	Apaïñiki	(st/ro/ba, to)	Bolivia (Chimane indians)	*Espundia* (cutaneous and mucocutaneous leishmaniasis)	^p^ *La*; ^*p*^ *Lb*; ^p^ *Ld*	Fournet et al. (1994)
			fresh-po (st, to)		*Espundia*	*L.*sp	Fournet et al. (1992a)
						*+*	
Polypodiaceae (2 quotes and 2 species)	*Campyloneurum angustifolium* Fée.	Calaguaça	(ss)	Ecuador	Cutaneous leishmaniasis	-	Weigel et al. (1994)
	*Phlebodium decumanum* (Willd.) J. Sm.	Coto Chupe	(rh)	Peru#	Uta	^p^ *Lm*	Kvist et al. (2006)
						IC_50_ > 100 μg/ml	
Phyllanthaceae (1 quote and 1 species)	*Phyllanthus attenuatus* Miq.	-	(le, to)	Ecuador#	-	-	Gachet et al. (2010)
Phytolaccaceae (1 quote and 1 species)	*Phytolacca dodecandra* L'Hér.	Endode (O,A)	“*The Root Is Powdered And Pasted With Butter”* pow-po (ro, to)	Ethiopia (Oromo)	Cutaneous leishmaniasis	-	Suleman and Alemu, (2012)
Picramniaceae (1 quote and 1 species)	*Picramnia* *sp*.	-	-	Colombia (Kofan)	Cutaneous leishmaniasis	-	Gutiérrez et al. (2014)
Pinaceae (1 quote and 1 species)	*Pinus* *sp*.	Piñón	(ss)	Ecuador	Cutaneous leishmaniasis	-	Weigel et al. (1994)
Piperaceae (13 quotes and 10 species)	*Piper aduncum* L.	Matico Chico	(le, to)	Bolivia	*Espundia* (cutaneous and mucocutaneous leishmaniasis)	^p^ *La*; ^*p*^ *Lb*; ^p^ *Ld*	Fournet et al. (1994)
	*Piper barbatum* Kunth.	-	(le, to)	Ecuador#	-	-	Gachet et al. (2010)
	*Piper consanguineum* (Kunth) Steud.	Matico	(le)	Ecuador	Cutaneous leishmaniasis		Weigel et al. (1994)
	*Piper hispidum* Sw. (2 quotes)	Atukan	fresh-po (le, to)	Peru (Chayahuita)	Uta	^p^ *La* = 69 μg/ml	Estevez et al. (2007)
						^a^ *La* = 5 μg/ml	
		-	(le, to)	Ecuador#	-	-	Gachet et al. (2010)
	*Piper loretoanum* Trel.	Atocan	pow-po (le, to)	Peru (Chayahuita)		^a^ *La* = 13.6 μg/ml	Odonne et al. (2009)
	*Piper mediocre* CDC.					-	
	*Piper musteum* Trel.	-	(le, to)	Ecuador#			Gachet et al. (2010)
	*Piper peltatum* L. (2 quotes	Sipu-sipu	(to)	Bolivia (Kechua)	*Espundia* (cutaneous and mucocutaneous leishmaniasis)	^p^ *La*; ^*p*^ *Lb*; ^p^ *Ld*	Fournet et al. (1994)
		-	(le, to)	Ecuador#	-	-	Gachet et al. (2010)
	*Piper umbellatum* L.	Amintë Huëron	fresh-po (le, to)	Peru (Chayahuita)	Ta’Ta’		Odonne et al. (2009)
	*Piper* *sp.* (2 quotes)	-	(le, to)	Ecuador#	-		Gachet et al. (2010)
		Atocan	pow-po (le, to)	Peru (Chayahuita)			Odonne et al. (2009)
					Ta’Ta’		
Plantaginaceae (3 quotes and 3 species)	*Conobea scoparioides* (Cham. & Schltdl.) Benth	Hierba De Sapo	(ae, to)	Colombia (Afro-Colombian and indigenous groups)	-	^p^ *La +*	Weniger et al. (2001)
						^p^ *Lb +*	
						^p^ *Li +*	
						^*a*^ *Lp +*	
	*Plantago major* L.	Llantén	(le)	Ecuador	Cutaneous leishmaniasis	-	Weigel et al. (1994)
	*Scoparia dulcis* L.	-	(le/wp, to)	Ecuador#	-		Gachet et al. (2010)
Poaceae (3 quotes and 3 species)	*Panicum trichoides* Sw	Lapakunga	-	Peru#	Uta or Chagas	^p^ *Lm* - NA	Kvist et al. (2006)
	*Pharus* *sp*.	-	(le, in)	Ecuador#	-	-	Gachet et al. (2010)
	*Zea mays* L.		(fl/le/fr, to)				
Polygonaceae (3 quotes and 3 species)	*Rumex nepalensis* Spreng.	Tult	fresh-po (ro/le, to)	Ethiopia	‘Gurtb’ leishmaniasis	-	Teklehaymanot (2009)
	*Rumex pulcher* L.	-	(le, to)	Ecuador#	-		Gachet et al. (2010)
	*Triplaris weigeltiana* (Rchb.) Kuntze.	Tangarana	(co)	Peru#	Uta or Chagas	^p^ *Lm*	Kvist et al. (2006)
						IC_50_ > 100 μg/ml	
Portulacaceae (1 quote and 1 species)	*Portulaca pilosa* L.	Tui	po (ae, to)	French Guiana (Wayãpi)	Leishmaniasis	-	Odonne et al. (2011a)
Primulaceae (1 quote and 1 species)	*Clavija weberbaueri* Mez.	-	(le, to)	Ecuador#	-	-	Gachet et al. (2010)
Pteridaceae (1 quote and 1 species)	*Pityrogramma calomelanos* (L.) Link.	Seseronapan	inf-bath (le, to)	Peru (Yanesha)	Uta De Agua, Mareñets	^a^ *La*	Valadeau et al. (2009)
						IC_50_ = 88 μg/ml	
Rhamnaceae (1 quote and 1 species)	*Gouania lupuloides* (L.) Urb.	-	(ba, to)	Ecuador#	-	-	Gachet et al. (2010)
Rosaceae (1 quote and 1 species)	*Prunus* *sp*.	-	(le/st, to)	Ecuador#	-	-	Gachet et al. (2010)
Rubiaceae (20 quotes and 15 species)	*Calycophyllum multiflorum* Griseb. (2 quotes)	Capirona	fresh-po (ba, to)	Peru	Uta		Vásquez-Ocmín et al. (2018)
		Quëmanan	pow-po (ba, to)	Peru (Chayahuita)	Ta’Ta’	^a^ *La*	Odonne et al. (2009)
						IC_50_ > 100 μg/ml	
	*Calycophyllum spruceanum* (Benth.) Hook.f. ex K.Schum.	Capirona	(co)	Peru#	Uta	^p^ *Lm -* NA	Kvist et al. (2006)
	*Capirona decorticans* Spruce (2 quotes)		inf-bath (ba, to)	Peru	Uta	-	Vásquez-Ocmín et al. (2018)
		Yoquinan		Peru (Chayahuita)	Ta’Ta’		Odonne et al. (2009)
		Llukina	“*The Bark Is Boiled And Watery Preparation Is Drunk Twice Dauly Until Cicatrisation”* dec (ba, or)		Uta	^p^ *La* - NA	Estevez et al. (2007)
						^a^ *La* IC_50_>100 μg/ml	
	*Coussarea* *sp*.	-	(ba, to)	Ecuador#	-	-	Gachet et al. (2010)
	*Genipa americana* L.	Isa	fresh-po (fr, to)	Peru (Chayahuita)	Ta’Ta’		Odonne et al. (2009)
	*Hamelia* *sp*.	-	(le, to)	Ecuador#	-		Gachet et al. (2010)
	*Kutchubaea cf. oocarpa* (Standl.) C.H.Perss.	Guayabochi	po (st/ba, to)	Bolivia#	Cutaneous leishmaniasis		Hajdu and Hohmann, (2012)
	*Ladenbergia* *sp*.	Quina, Miraña, Guayabate, Resbalomono, Sicomue (Col.)	-	Colombia (Kofan)	Cutaneous leishmaniasis		Gutiérrez et al. (2014)
	*Palicourea* *sp*.	-					
	*Psychotria* *sp*. (4 quotes)		(le, to)	Ecuador#	-		Gachet et al. (2010)
			(le/st, to)				
			(le, to)				
		Beso Rojo	-	Colombia (Kofan)	Cutaneous leishmaniasis		Gutiérrez et al. (2014)
		-					
	*Rudgea bremekampiana* Steyerm.		(le, to)	Ecuador#	-		Gachet et al. (2010)
	*Rudgea loretensis* Standl.	Niahuënara	fresh-po (ba, to)	Peru (Chayahuita)		^a^ *La*	Odonne et al. (2009)
						IC_50_ = 34–39.6 μg/ml	
	*Spermacoce laevis* Lam.	-	(le/st, to)	Ecuador#		-	Gachet et al. (2010)
	*Uncaria guianensis* (Aubl.) J.F.Gmel.	Ochara	(or/to)	Peru (Chayahuita)	Ta’Ta’		Odonne et al. (2009)
	*Uncaria tomentosa* (Willd. ex Schult.) DC.		-		Cutaneous leishmaniasis		
Rutaceae (12 quotes and 7 species)	*Angostura longiflora* (K.Krause) Kallunki.	Evanta	(le/st/ro, to)	Chimane indians (Bolivia)	*Espundia* (cutaneous and mucocutaneous leishmaniasis)	^p^ *La*; ^*p*^ *Lb*; ^p^ *Ld*	Fournet et al. (1994)
	*Citrus aurantiaca* (L.) Swingle.	Limón	-	Ecuador	Cutaneous leishmaniasis	-	Weigel et al. (1994)
	*Citrus × aurantiifolia* (Christm.) Swingle	Nimo	fresh-po (fr/ba, to/in)	Peru (Chayahuita)	Ta’Ta’, Huayani		Odonne et al. (2009)
	(2 quotes)	Citron Vert	inf-po (fr, to)	French Guiana (Wayãpi)	Leishmaniasis		Odonne et al. (2011a)
	*Citrus × aurantium* L. (3 quote)	Naranja	-	Ecuador	Cutaneous leishmaniasis		Weigel et al. (1994)
		Toronja	(ro)	Peru#	Uta	^p^ *Lm*	Kvist et al., (2006)
						IC_50_ = 95 μg/ml	
		Mandarina	-	Ecuador	Cutaneous leishmaniasis	-	Weigel et al. (1994)
	*Citrus × limon* (L.) Osbeck.	Lìmón	(ro)	Peru#	Uta	^p^ *Lm*	Kvist et al. (2006)
						IC_50_ = 70 μg/ml	
	*Citrus* *sp.* (2 quotes)	-	(se, to)	Ecuador#	-	-	Gachet et al. (2010)
			(fr, to)				
	*Ruta graveolens* L. (2 quotes)		(le/fr, to)				
		Ruda	(le)	Ecuador	Cutaneous leishmaniasis		Weigel et al. (1994)
Sapindaceae (1 quote and 1 species)	*Dodonaea viscosa* Jacq.	Shath	po (le, to)	Saudi Arabia	Leishmaniasis	-	Ali et al. (2017)
Sapotaceae (7 quotes and 6 species)	*Chrysophyllum prieurii* ADC.	Cotoquinilla	fresh-po (le, to)	Peru	Uta	-	Vásquez-Ocmín et al. (2018)
	*Chrysophyllum* *sp*.	-	(se, to)	Ecuador#	-		Gachet et al. (2010)
	*Manilkara* *sp*.	Baytakini	inf-bath, lt, to	French Guiana (Teko)	Leishmaniasis		Odonne et al. (2011a)
	*Pouteria caimito* (Ruiz & Pav.) Radlk.	Caimito	(le)	Peru#	Uta	^p^ *Lm*	Kvist et al. (2006)
						IC_50_ > 100 μg/ml	
	(2 quotes)	Guëpa	fresh-po (le, to)	Peru (Chayahuita)	-	-	Odonne et al. (2009)
	*Pouteria guianensis* Aubl.	Caimito		Peru	Uta		Vásquez-Ocmín et al. (2018)
	*Pouteria torta subsp. tuberculata* (Sleumer) T.D.Penn.	-	(le, to)	Ecuador#	-		Gachet et al. (2010)
Siparunaceae (3 quotes and 1 species)	*Siparuna* *sp.* (3 quotes)	Huaya Muuktuna	*“The Woody Stem Grated Ad Boiled. This Preparation Is Drunk Three Times A Day For 8 Days”* dec (st, or)	Peru (Chayahuita)	Uta	^p^ *La*	Estevez et al. (2007)
						IC_50_ = 30 μg/ml	
						^a^ *La* IC_50_>100 μg/ml	
		Huayan Motonan	fresh-po (le, to)		-	-	Odonne et al. (2009)
Smilacaceae (4 quotes and 2 species)	*Smilax salicifolia* Griseb.	Sankarin	*“Roots Are Boild, And This Preparation Is Drunk Many Times A Day, Until Symptoms Disappear”* dec (ro, or)	Peru (Chayahuita)	-	^p^ *La* - NA	Estevez et al. (2007)
						^a^ *La* IC_50_>100 μg/ml	
	*Smilax* *sp.* (3 quotes)	-	(wp, to)	Ecuador#		-	Gachet et al. (2010)
		Zarzaparilla	(ro)	Peru#	Uta or Chagas	^p^ *Lm*	Kvist et al. (2006)
						IC_50_ > 100 μg/ml	
			(le)	Ecuador	Cutaneous leishmaniasis	-	Weigel et al. (1994)
Solanaceae (22 quotes and 14 species)	*Brugmansia* *sp.* (2 quotes)	-	(le/fl, to)	Ecuador#	-	-	Gachet et al. (2010)
			(le, to)				
	*Brunfelsia grandiflora* D.Don. (3 quotes)	Ohuinishqui	pow-po (le, to/in)	Peru (Chayahuita)	Ta’Ta’, Huayani		Odonne et al. (2009)
		-	(le, to)	Ecuador#	-		Gachet et al. (2010)
		Chiric Sanango	(ro)	Peru#	Uta	^p^ *Lm*	Kvist et al. (2006)
						IC_50_ = 53 μg/ml	
	*Capsicum* *sp.* (2 quotes)	-	(le, to)	Ecuador#	-	-	Gachet et al. (2010)
		No’Ca	fresh-po, (le/fr, to)	Peru (Chayahuita)	Ta’Ta’, Huayani	^a^ *La*	Odonne et al. (2009)
						IC_50_ = 28 μg/ml	
	*Cestrum lindenii* Dunal.	-	(le, to)	Ecuador#	-	-	Gachet et al. (2010)
	*Cestrum* *sp*.		-	Colombia (Kofan)	Cutaneous leishmaniasis	-	Gutiérrez et al. (2014)
	*Markea* *sp*.		(le, to)	Ecuador#	-		Gachet et al. (2010)
	*Nicotiana tabacum* L. (3 quotes)		(le, to)				
		Pinchi	pow-po (le, to/in)	Peru (Chayahuita)	Ta’Ta’, Huayani		Odonne et al. (2009)
		Tabaco	fresh-po (le fermented, to)	French Guiana	Leishmaniasis		Odonne et al. (2011a)
	*Solanum americanum* Mill. (2 quotes)	-	(wp, to)	Ecuador#	-		Gachet et al. (2010)
		Yerba Mora (Mortino)	(fr/le)	Ecuador	Cutaneous leishmaniasis		Weigel et al. (1994)
	*Solanum crinitum* Lam.	Y˜ U ãsisi	po (ba, to)	French Guiana (Wayãpi)	Leishmaniasis		Odonne et al. (2011a)
	*Solanum incanum* L.	Al-hadak	po	Saudi Arabia			Ali et al. (2017)
	*Solanum mammosum* L.	-	(le, to)	Ecuador#	-		Gachet et al. (2010)
	*Solanum subinerme* Jacq.	Y˜ U Sõwú	po (ba, to)	French Guiana (Wayãpi)	Leishmaniasis		Odonne et al. (2011a)
	*Solanum* *sp.* (2 quotes)	-	(le, to)	Ecuador#	-		Gachet et al. (2010)
			(fr, to)				
	*Witheringia solanacea* L'Hér.		(le/st/wp, to)				
Talinaceae (1 quote and 1 species)	*Talinum paniculatum* (Jacq.) Gaertn.	Yoro Qui’Sha	fresh-po (ro, to)	Peru (Chayahuita)	Ta’Ta’	^a^ *La* IC_50_>100 μg/ml	Odonne et al. (2009)
Thurniaceae (1 quote and 1 species)	*Thurnia sphaerocephala* (Rudge) Hook.f.	Kwayiti	(fr, to)	French Guiana (Teko)	Leishmaniasis	-	Odonne et al. (2011a)
Ulmaceae (1 quote and 1 species)	*Ampelocera edentula* Kuhlm.	Sou’Sou’	(st/ro, to)	Bolivia (Chimane indians)	*Espundia* (cutaneous and mucocutaneous leishmaniasis)	^p^ *La*; ^*p*^ *Lb*; ^p^ *Ld*	Fournet et al. (1994)
Urticaceae (3 quotes and 3 species)	*Cecropia obtusa* Trécul.	Ama’I	fresh-po (ba, to)	French guiana (wayãpi)	Leishmaniasis	-	Odonne et al. (2011a)
	*Urera laciniata* Wedd.	-	(le/st, to)	Ecuador#	-		Gachet et al. (2010)
	*Urtica dioica* L.		(le, to)				
Verbenaceae (8 quotes and 6 species)	*Duranta* *sp*.	-	(le, to)	Ecuador#	-	-	Gachet et al. (2010)
	*Lantana camara* L.						Gachet et al. (2010)
	*Lantana trifolia* L.	Yahua’Tan Huëron	pow-po (le, to)	Peru (Chayahuita)			Odonne et al. (2009)
	*Lantana* *sp.* (3 quotes)	T Epeshpan	inf-po (le, to)	Peru (Yanesha)	Uta De Agua, Mareñets	^a^ *La*	Valadeau et al. (2009)
						IC_50_ = 10 μg/ml	
					Leishmaniasis	-	Valadeau et al. (2010)
		-	(le/st, to)	Ecuador#	-		Gachet et al. (2010)
	*Verbena litoralis* Kunth.		(le/st/wp, to)				
	*Verbena microphylla* Kunth.	Berbena/Verbena	(le)	Ecuador	Cutaneous leishmaniasis	-	Weigel et al. (1994)
Viburnaceae (1 quote and 1 species)	*Sambucus nigra* L.	-	(le/st, to)	Ecuador#	-	-	Gachet et al. (2010)
Violaceae (1 quote and 1 species)	*Leonia* *sp*.		(le, or/to)	Ecuador#	-	-	Gachet et al. (2010)
Zamiaceae (3 quotes and 3 species)	*Zamia amazonum* D.W.Stev.	Oreja De Perro	fresh-po (ro, or/to)	Peru (Chayahuita)	Uta	^p^ *La*>100 μg/ml	Estevez et al. (2007)
						^a^ *La* = 81 μg/ml	
	*Zamia poeppigiana* Mart. & Eichler.	Ukuapampe	fresh-po (st, to)			^p^ *La*>100 μg/ml	
						^a^ *La* = 33 μg/ml	
	*Zamia* *sp*.	Ocohua Panp	fresh-po (ba, to)		-	-	Odonne et al. (2009)
Zingiberaceae (2 quotes and 1 species)	*Zingiber officinale* Roscoe. (2 quotes)	Natio	fresh-po (rh, to/in)	Peru (Chayahuita)	Ta’Ta’, Huayani	-	Odonne et al. (2009)
		-	(wp, to)	Ecuador#	-		Gachet et al. (2010)

Traditional recipe: Decoction—dec; Decoction used as a poultice - dec-po; Decoction used as a bath—dec-bath; Fresh plant used as a poultice—fresh-po; Infusion—inf; Infusion used as a bath—inf-bath; Infusion used as a poultice—inf -po; Poultice—po; Powder plant used as a poultice—pow-po. Plant Part: Aerial Part—ae; Apical meristem—am; Bark—ba; Bulb—bu; Cloves—cl; Cortex—co; Exudate—ex; Flower—fl; Fruit—fr; Leaf -le; Látex—lt; Oleogum Resin—or; Resin - re; Rhizome—rh; Root—ro; Sap—sa; Seed—se; Shoot—sho; Stalk—sta; Stem—st; Tuber—tu; Whole Plant -wp. Route: Inhalation—in; Nasal—na; Oral—or; Topical—to. Countries and Communities: Ecuador# - Kichwa of Amazonia, Kichwa of the Andes, Chachi, Mestizo, Afroecuadorian, Awa, Épera; Bolivia# - Guarasug'we indigenous and Chiquitano mestizos; Peru# - Mestizo, Chayahuito, Cocama, Quechua, Ticuna. ^p^promastigote; ^a^amastigote; *La*—*L. amazonensis*; *Lae*—*L. aethiopica; Lb*—*L. braziliensis Lp*—*L. panamensis; L. donovani; Lm*—*L. major; Li*—*L.infantum;* IC_50_—Inhibitory concentration 50%; NA—Not active.

* In the above citations we counted each species and genus as a single citation, for example, in the case of the genus *Gurania* sp. although it was cited two times in the articles, it was accounted as one species, because it was not possible to classify *Gurania* sp. as one or two species. Additionally, it is not possible to know if these two quotes of the genus *Gurania* in this table belong to *Gurania lobate* (L.) Pruski, because taxonomic elements are lacking in the published articles. Thus, the 292 plants here presented refer to 216 species (identified until species level) and 76 genera (the ones counted only once).

**TABLE 2:** *In vivo* activity of medicinal plants. Families, plant species, clinical form of leishmaniasis, parasite species, extract or purified molecules employed in experimental treatment, doses, route of administration, scheme of treatment and efficacy of the treatments in experimental leishmaniasis.

Family	Plant species	Clinical form and parasite species	Treatment	Route of administration	Efficacy	Ref
Amaranthaceae	*Dysphania ambrosioides* (L.) Mosyakin & Clemants	CL – *L.a*	Essential oil (30 mg/kg)	Intraperitoneal, once a day for 15 days	Reduced by ∼68% the number of parasites	Monzote et al. (2006)
			Leaves - hydroalcoholic crude extract (5 mg/kg)	Intralesional, 5 injection at every 4 days	Intralesional: Reduced parasitism by ∼66, 95, 66% in the skin, lymph nodes and spleen	Patrício et al. (2008)
				Oral, once a day for 15 days	Oral: No effect	
Amaryllidaceae	*Allium sativum* L.	CL—*L.m*	Fresh garlic bulb—aqueous extract (20 mg/kg)	Intraperitoneal, daily for 15 days	Reduced by ∼ 65% the size of cutaneous lesions	Ghazanfari et al. (2000)
			Fresh and dried garlic bulb—aqueous extract (20 mg/kg)	Intraperitoneal, daily for 15 days	Dried extract—inhibited lesion progression and parasite multiplication	Gamboa-León et al. (2007)
					Fresh extract—No effect	
		CL—*L.m*	Fresh garlic bulb—methanolic extract (20 mg/kg)	Oral and intraperitoneal, daily for 4 weeks	CL—oral and intraperitoneal treatment reduced by ∼90 and 80% the size of skin lesion, respectively	Wabwoba et al. (2010)
		VL—*L.d*			VL—oral and intraperitoneal treatments reduced by ∼65 and 55% the number of splenic parasites, respectively	
Apocynaceae	*Pentalinon andrieuxii* (Müll.Arg.) B.F.Hansen & Wunderlin	CL—*L.me*	Root hexanic extract (10 μg)	Topical; once a day for 6 weeks	Reduced in 2 times the number of parasites in the skin	Lezama-Dávila et al. (2014)
		VL—*L.d*	Liposome-encapsulated pentalinonsterol (2.5 mg/kg)	Intravenous	Reduction of 64, 83 and 57% of parasites in the liver, spleen and bone marrow, respectively	Gupta et al. (2015)
	*Tabernaemontana divaricata* (L.) R.Br. ex Roem. & Schult.	VL—*L.d*	Voacamine (2.5—5 mg/kg)	Intraperitoneal; twice a week for three weeks	*Hepatic parasitism*	Chowdhury et al. (2017)
					2.5 and 5 mg/kg: decreased in ∼3 and 30 times the tissue parasitism, respectively	
					*Splenic parasitism*	
					2.5 and 5 mg/kg: decreased in ∼5 and 15 times the tissue parasitism, respectively	
Asteraceae	*Munnozia maronii* (André) H.Rob	CL—*L.a*	Dehydrozaluzanin C (100 mg/kg)	Once a day for 14 days	Reduced the severity of cutaneous lesions	Fournet et al. (1993b)
Bignoniaceae	*Handroanthus serratifolius* (Vahl) S.O.Grose	CL—*L.a*	Lapachol (25 mg/kg)	Oral; once a day for 10 days	CL—reduction of ∼24.5 times the number of parasites	Araújo et al. (2019)
		VL—*L.i*			VL—reduction of ∼4.6 and 5.3 the number of parasites in the spleen and liver, respectively	
Euphorbiaceae	*Croton caudatus* Geiseler	LV—*L.d*	Leaves - semi purified fraction (1.25; 2.5; 3.75; and 5 mg/kg)	Oral; five consecutive days	*Hepatic parasitism*	Dey et al. (2015)
					2.5, 3.75 and 5 mg/kg reduced the parasitism by ∼ 40, 60, and 65%, respectively	
					*Splenic parasitism*	
					1.25; 2.5, 3.75, and 5 mg/kg reduced the parasitism by 36.2, 51.7, 66.71 and 69.12%, respectively	
Fabaceae	*Pleurolobus gangeticus *(L.) J.St.-Hil. ex H.Ohashi & K.Ohashi	VL—*L.d*	Whole plant - ethanolic extract; hexane; n-butanol and aqueous fractions (100 mg/day)	Oral route, once a day for 5 consecutive days	Animals treated with n-butanol fraction reduced the number of splenic parasites by 46.7%	Singh et al. (2005)
	*Copaifera martii* Hayne	CL—*L.a*	Copaiba oil (100 mg/kg)	Subcutaneous; oral; topical; oral + topical; for 4 weeks	Oral, oral plus topical treatments decreased the lesion size by ∼ 4 times	dos Santos et al. (2011)
Piperaceae	*Piper rusbyi* C. DC.	CL—*L.a*	Flavokavain B (1–5 mg/kg)	Subcutaneous, alternative days for 28 days	Animals treated with 5 mg/kg displayed reduction in the size of lesions by 32.2%	Flores et al. (2007)
	*Piper pseudoarboreum* Yunck	CL—*L.a*	(E)-piplartine	Intralesional, once a day for 4 days	Reduction of skin lesions and visceralization by 35 and 55%, respectively	Ticona et al. (2020)
			(25 mg/kg)			
Rutaceae	*Angostura longiflora* (K.Krause) Kallunki	CL—*L.a*	Root and stem bark- total alkaloid extract (50 mg/kg)	Oral, twice daily for 15 days	Root extract: Oral and intralesional treatments reduced the parasite load by 95 and 96%, respectively	Fournet et al. (1996)
				Intralesional, five times at intervals of 5 days	Stem extract: Intralesional and oral treatments decreased the parasite loads by 99 and 49%, respectively	
		CL—*L.b*	Bark - total alkaloid extract (12.5mg/animal)	Intraperitoneal, once a day until the week 14	Reduced in ∼10 times the number of parasites	Calla-Magariños et al. (2013)
Solanaceae	*Solanum lycocarpum* A.St.-Hil	CL—*L.me*	Solamargine plus solasonine (10 μg)	Topical, once a day for 6 weeks	Reduced in 3 times the number of parasites	Lezama-Dávila et al. (2016)
	*Solanum havanense* Jacq.*, Solanum myriacanthum* Dunal*, Solanum nudum* Humb. & Bonpl. ex Dunal*, Solanum seaforthianum* Andrews	CL—*L.a*	Leaves—hydroalcoholic extracts (30 mg/kg)	Intralesional, every 4 days, 5 doses	Reduction of parasites in animals treated with	Cos et al. (2018)
					*S. havanense* (93.6%)*, S. nudum* (80%) *S. myriacanthum* (56.8%) and *S. seaforthianum* (49.9%)	
Urticaceae	*Urtica thunbergiana* Siebold & Zucc*.*	CL—*L.m*	Plant aqueous extract (150; 200, and 250 mg/kg)	Intramuscular and intralesional, three times/week for 30 days	All treatments inhibited lesion development and suppressed parasite dissemination, with special activity to the intralesional treatment	Badirzadeh et al. (2020)

CL—Cutaneous leishmaniasis; VL—visceral leishmaniasis; L.a—Leishmania (Leishmania) amazonensis; L.d—Leishmania (Leishmania) donovani; L.i—Leishmania (Leishmania) infantum; L.m—Leishmania (Leishmania) major; L.me—Leishmania (Leishmania) mexicana; L.b—Leishmania (Viannia) braziliensis.

Table 1 summarizes the findings observed in the ethnopharmacological surveys and contains the following data: family, scientific and vernacular names, traditional recipe (part of plant used and route), country (traditional community involved in the knowledge), traditional use (emic term, the one used by the communities), and whether the study included laboratory assays to determine the efficacy of plant extracts on *Leishmania* sp.

The map (Figure 2) was prepared using the software QGIS (available at www.qgis.org) using a collection of spatial data from the Brazilian Institute of Geography and Statistics (available at https://mapas.ibge.gov.br/bases-e-referencial/bases-cartograficas/digital meshes) and using the geographic coordinates reference system "sirgas 200" (Geocentric Reference System for the Americas).

## Plants Recommended for the Treatment of leishmaniasis by traditional Communities Worldwide

### Plants (species, families and vernacular names)

From the 20 selected articles, 378 quotes were obtained referring to 292 plants indicated by several traditional communities around the world to treat leishmaniasis. These plants belong to 89 taxonomic families (Table 1). To record the number of plants, each species and genus was counted as a single citation; for example, in the case of the genus *Gurania* sp. Although it was cited two times in the articles, it was considered one species because it was not possible to classify *Gurania* sp. as one or two species. Additionally, it is not possible to know if these two examples of the genus *Gurania* belong to *Gurania lobate* (L.) Pruski—as illustrated in Table 1 - because taxonomic elements were not available in the articles. Thus, the 292 plants presented herein refer to 216 species (identified until the species level) and 76 genera (the ones counted only once) (Table 1). Only 74% of the plants available in the articles could be identified to the species level, pointing out the need for more adequate ethnopharmacology methods during fieldwork.

Considering those 378 plant quotes, the most frequent families used by traditional communities were Fabaceae (27 quotes); Araceae (23); Asteraceae and Solanaceae (22 each), Euphorbiaceae (21) and Rubiaceae (20) (Figure 1).

Moreover, 207 out 292 plants had their vernacular names (Table 1) described in the publications. The absence of these data makes ethnopharmacological analysis precarious, since recording the vernacular name of a certain plant can provide valuable information about its potential pharmacological effects. An example discussed by us in a previous work is the plant *caprankohirehô* (Euphorbiaceae), which has been used by the Brazilian Krahô Indians as a tranquilizer, and the literal translation of *caprankohireho* is the ‘leaf of turtle spine’. This translation describes the pharmacological effect of this plant—which induces ‘slowness’ (Rodrigues and Barnes, 2013). This and many other examples demonstrate that the careful recording of vernacular names of plants during ethnopharmacological studies is extremely relevant to increase the probability of finding bioactive molecules according to the knowledge of traditional communities.

In addition, from the 216 plants described up to the species level, only 29 were present in at least two articles; six out 29 species were described in three articles: *Brunfelsia grandiflora* D. Don (Solanaceae), *Capirona decorticans* Spruce (Rubiaceae), *Chelonanthus alatus* (Aubl.) Pulle (Gentianaceae), *Hura crepitans* L. (Euphorbiaceae), *Nicotiana tabacum* L. (Solanaceae), *Tabernaemontana sananho* Ruiz & Pav. (Apocynaceae), while the following four species were cited in four articles: *Carica papaya* L. (Caricaceae), *Cedrela odorata* L. (Meliaceae), *Copaifera paupera* (Herzog) Dwyer (Fabaceae), and *Musa × paradisiaca* L. (Musaceae) (Table 1).

In Table 1, it was also observed that most of the species were cited by traditional communities from only one country, 26 species were cited by at least two countries. Three of them belonged to the traditional communities of Peru, Ecuador, and French Guiana simultaneously: *Carica papaya* L. (Caricaceae), *Musa × paradisiaca* L. (Musaceae), and *Nicotiana tabacum* L. (Solanaceae).

### Recipes (parts of the plants used, method of preparation, route of administration)

As registered in Table 1, not all ethnopharmacological studies gave information on the parts of the plants used, the form of preparation, route of administration, dose, and/or duration of the treatment. Considering the 378 quotes, only 138 (36.5%) specified the recipes, 351 (92.9%) mentioned the plant parts used in the recipe, and 300 (79.4%) detailed the routes of administration of the recipes. The absence of these data offers two possible justifications. The first possible explanation may be the lack of adequate methods during ethnopharmacological fieldwork; although this may be less likely, such works may reflect the lack of knowledge of these data on the part of the communities under study. The absence of these data can impact further studies on phytochemistry and pharmacology and, as a consequence, the discovery of new bioactive molecules of medicinal plants. On the other hand, several ethnopharmacological studies described in great detail the recipes used in the treatment of leishmaniasis. An example is the study conducted by Vásquez-Ocmín and collaborators (Vásquez-Ocmín et al., 2018), which described the use of the plant *Virola surinamensis* (Rol. ex Rottb.) Warb. (Myristicaceae), whose popular name is Cumala Colorada (Table 1). The bark was used as described by the interviewee during the field work *“… Boiled 5 g of the bark in 1 L of water. Drink one cup every morning for three days … ”.* In other words, all necessary information was offered in detail, except for possible contraindications and adverse events of the plant.

Among the available data in the 378 quotes, it was observed that the parts of the plants most frequently used in local medicine were leaves (42.3% of recipes), followed by bark (15%), stems (11.6%), and roots (5.6%). On the other hand, the fruits, aerial parts, flowers, oleoresins, seeds, tubers, whole plants, stalks, shoots, saps, resin, rhizomes, apical meristems, bulbs, cloves, exudates and latex were used at minor frequencies. The most suitable route of administration for plants was the topical route (74.6% of the recipes), followed by the oral route (5%) and inhalation/nasal route (1.3%); for a large number of plants, no route of administration was indicated (20.6%).

In addition, as shown in Table 1, 17.2% of the methods used to prepare the recipes refer to fresh poltices (lotion juice in natura, crushed, crude parts, paste) applied on the affected area, named fresh-po in Table 1, followed by pow-po (6.3%), which are powered plants that are also applied on the wounds. Finally, with minor frequencies, other methods were mentioned, such as decoction and infusion that can be ingested and/or used to wash the affected area. In these last cases, they were presented in Table 1 as inf-po (infusion used as a poltice) and dec-po (decoction used as a poltice).

In the selected studies, a predominance of leaves (42.3%) used topically (74.6%) for the treatment of leishmaniasis was observed. Several studies, including those carried out by some members of our team, point out the use of leaves and the topical route in traditional treatments for leishmaniasis. Thus, the quilombolas in the Pantanal from Poconé, Brazil, use a decoction-type tea with the leaf/bark of mangava-brava—*Lafoensia pacari* A. St.-Hil. (Lythraceae) to be ingested twice a day; the juice from the leaves of mastruz, *Dysphania ambrosioides* (L.) Mosyakin & Clemants (Amaranthaceae), is used as a compress to treat leishmaniasis; finally, the river dwellers from Amazon, Brazil, use the bark of mango, *Mangifera indica* L. (Anacardiaceae), as a compress directly on the cutaneous lesions (Rodrigues, 2006).

### Knowledge of traditional communities in the world

The analysed works showed that traditional communities spread across seven countries use plants for the treatment of leishmaniasis. The majority of these communities are located in Latin America. Ecuador is the most representative of the range of plants indicated in the treatment of leishmaniasis (59 botanical families; 145 plant species; seven traditional communities; two articles), followed by Peru (39; 80; 8; 7), French Guiana (22; 34; 2; 1), Bolivia (15; 20; 7; 4) and Colombia (14; 16; 2; 2). In addition to these countries, studies developed in Saudi Arabia (8; 8; 1; 1) and Ethiopia (6; 6; 2; 3) (Figure 2) also highlighted the use of medicinal plants in the treatment of leishmaniasis.

Brazil and Colombia are countries with a high occurrence of cases of cutaneous leishmaniasis, above five thousand. However, the data collected show few or no published studies involving the use of traditional knowledge for the treatment of this infectious disease, with only two studies found in Colombia and none in Brazil. Although during this review it was not possible to obtain Brazilian studies focusing on “ethnopharmacology x leishmaniasis”, some studies within the scope of ethnopharmacology have offered information on the use of natural resources for the treatment of leishmaniasis (França et al., 1996; Rodrigues, 2006; Santos et al., 2019), but they were not included in this review, as they were not found during the Boolean search.

Figure 2 (a) highlights in yellow the endemic countries that had more than five thousand cases of cutaneous leishmaniasis until 2018 (World Health Organization, 2019). In part (b) of Figure 2, emphasis was given to the numbers of botanical families and species, articles, and traditional communities that contributed to ethnopharmacological research in each of the countries of Latin America, since these were the most expressive when considering the data on traditional knowledge *vs.* leishmaniasis.

The data on the traditional communities that participated in the studies analyzed herein exhibited the relevant contribution of traditional knowledge from South America in the treatment of leishmaniasis, and this is correlated with the continent that displays the highest number of cases of cutaneous leishmaniasis in the world, suggesting that in some areas, medical services are not available, and people need to use alternative medicines. Figure 2 shows the amount of data associated with the traditional treatment of leishmaniasis generated by traditional communities in countries with a high incidence of leishmaniasis. Of all countries with cases of cutaneous leishmaniasis, only 40% also presented ethnopharmacological studies on the disease. Among them, the country that presented the most studies was Peru (7 studies), followed by Bolivia (4). Both are low-income countries, with deficiencies in their economic and educational systems. The main traditional communities cited among the analyzed articles belong to the following ethnic groups from Ecuador: Kichwa of Amazonia, Kichwa of the Andes, Chachi, Mestizo, Afroecuadorian, Awa and Épera (contributing 38.3% of the citations of plants to treat leishmaniasis), followed by Peruvian ethnic groups Chayahuita (22.7%), Wayãpi of French Guiana (7.6%) and Yanesha of Peru (5.5%). In addition, 12.9% of the citations did not mention the community that provided traditional knowledge, and some of the authors referred to them as local people or ethnic groups. In relation to the total number of studies analyzed, two out seven countries (Ethiopia and Saudi Arabia) had no record of the occurrence of cutaneous leishmaniasis above 5,000 cases. According to World Health Organization (2019), both Ethiopia and Saudi Arabia had a record of 100–999 cases of cutaneous leishmaniasis.

It is important to note that leishmaniasis exhibits different clinical forms that can be recognized and named in different ways depending on the specificity of each country and ethnic group. In ethnopharmacological studies, the correlation between the emic terms (the ones used by the traditional communities) and their corresponding etic terms (the ones used in biomedicine) may provide insights to guide further pharmacological studies since they are the bases for suggesting the potential bioactivity of these resources (Pagani et al., 2017). Approximately half of the articles present records of emic terms to leishmaniasis, such as “Gurtb”, in Ethiopia (Teklehaymanot, 2009); “Espundia” for the Chimane Indians, in Bolivia (Fournet et al., 1992b, 1994); “Ta’Ta’ ”, for the Chayahuitas in Peru (Odonne et al., 2009); “Uta” and “Uta De Agua” for some communities in Peru, such as Chayahuitas or Yaneshas (Estevez et al., 2007; Valadeau et al., 2009; Vásquez-Ocmín et al., 2018).

### Plants tested for leishmaniasis

From the 292 plants registered, 79 described in nine of the twenty selected articles were tested against *Leishmania* sp. Among the *Leishmania* species investigated in these studies*, L. (L.) amazonensis* predominated, followed by *L. (L.) major* and *L. (V.) braziliensis*. The results of the tests with some of these plants are available in more than one publication, including the resins and saps of *Copaifera paupera* (Herzog) Dwyer and the bark and cortex of *Spondias mombin* L. (Kvist et al., 2006; Estevez et al., 2007), the latex and resin of *Hura crepitans* L. (Fournet et al., 1994; Kvist et al., 2006), the stem bark and root bark of *Pera benensis* Rusby (Fournet et al., 1992a, 1994), and the leaves of *Pseudelephantopus spicatus* (B. Juss. ex Aubl.) Rohr ex C.F. Baker (Odonne et al., 2009; 2011b). Below, descriptions of the *in vitro* activity of extracts or purified molecules from the plants used in traditional communities will be provided.

Estevez and colleagues (Estevez et al., 2007) investigated the leishmanicidal activity of nineteen plants indicated by the Chayahuite community to treat cutaneous leishmaniasis. Among them, only the ethanolic extracts produced with the leaves of *Piper hispidum* Sw. and *P. strigosum* Trel (Piperaceae) showed expressive activity against intracellular forms of *L. (L.) amazonensis*.

Odonne and collaborators (Odonne et al., 2009) observed that different plants have been used by the Chayahuites in the treatment of leishmaniasis, probably because they live in an endemic area of the disease and have limited access to medical centers. The leishmanicidal activities of ethanolic extracts produced with the selected plants were evaluated in axenic amastigote forms of *L. (L.) amazonensis*. Ethanolic extracts produced with the aerial parts of *Desmodium axillare*, *Pseudoelephantopus spicatus* and *Piper loreteanum* were the most active extracts at eliminating amastigote forms (IC_50_ between 13.6 and 27 μg/ml). Ethanolic extracts produced with the bark and/or leaves of *Rudgea loretensis* Standl and *Salacia juruana* Loes showed moderate leishmanicidal activity (IC_50_ between 34 and 41 μg/ml). In addition, all these plants were clearly indicated to treat leishmaniasis. On the other hand, it was also demonstrated that ethanolic extracts produced with plants that have not been used to treat leishmaniasis showed significant leishmanicidal activity (IC_50_ between 10 and 15.7 μg/ml), as is the case for ethanolic extracts produced with the leaves, roots and aerial parts of *Piper sanguineispicum* Trel., *Cybianthus anthuriophyllus* Pipoly, (Myrsinaceae), *Clibadium sylvestre* (Aubl.) Baill. (Asteraceae), respectively.

Further studies characterize the major components in the ethanolic extract produced with the leaves of *Pseudoelephantopus spicatus*. The purified molecules 1) 8,13-diacetyl-piptocarfol, 2) 8-acetyl-13-O-ethyl-piptocarfol [also isolated from other species: *Vernonia mollissima* (D. Donex Hook. & Arn.), *Eirmocephala megaphylla* (Hieron.) H. Rob., *Chrysolaena verbascifolia*, *Lepidaploa remotiflora,* and *Vernonia scorpioides*] and 3) ursolic acid (Odonne et al., 2011b) were assayed on axenic amastigote forms of *L. (L.) amazonensis*. Molecules 1 (IC_50_ = 0.2 μM) and 2 (IC_50_ = 0.37 μM) showed leishmanicidal activity (*in vitro*) comparable with amphotericin B (IC_50_ = 0.41 μM), which is used in the treatment of human leishmanial infections. Molecule 3 also eliminated amastigote forms with high activity (IC_50_ = 0.99 μM). Although leishmanicidal action has been observed, the authors considered that the second compound originated from the chemical reaction resulting from the extraction of the ethanolic extract and not from the plant in natura. This work corroborated the leishmanicidal effects observed during traditional treatment (Odonne et al., 2009; 2011b); in addition, it showed for the first time the production and accumulation of such classes of secondary metabolites in *P. spicatus* and supported further preclinical works with molecule 3 in the context of cutaneous and visceral leishmaniasis (Jesus et al., 2020; de Jesus et al., 2021), which in fact reinforces the occurrence of important bioactive molecules in plants traditionally used to treat leishmaniasis.

In the community of Buena Vista, Bolivia, thirty-eight plants have been used to treat skin problems, and eight of them were recommended by Tacana medicine for the treatment of leishmaniasis (Arévalo-Lopéz et al., 2018). Extracts were produced with all these plants, and the leishmanicidal activity assayed on promastigote forms of *L. (L.) amazonensis* and *L. (V.) braziliensis.* It was observed that 42.1% of them were inactive and 23.7% highly active, and the leishmanicidal activity of 34.2% of them was dependent on the part of plant used to produce the extracts. With respect to the plants that were specifically indicated to treat leishmaniasis, extracts produced with the leaves of *Hyptis mutabilis* (Laminaceae) and the bark of *Jacaranda glabra* (Bignoniaceae) and *Tessaria integrifolia* (Asteraceae) were active on *L. (L.) amazonensis* and *L. (V.) braziliensis*. Further studies showed that fractions purified from the crude ethanolic extracts of *J. glabra* and *T. integrifolia* were also active toward promastigote forms of *L. (L.) amazonensis*, *L. (L.) aethiopica*, *L. (V.) braziliensis* and *L. (V.) lainsoni*. Although extracts and fractions produced with these plants displayed multispecies action, it was noted that the selective indexes of these natural medicines were low when compared with amphotericin B. On the other hand, it is relevant to observe that in the field, the production of these natural medicines is completely different from those produced in the laboratory, and it can account for the extraction of cytotoxic molecules. Furthermore, this study showed the leishmanicidal activity of five species of Tacana medicinal plants for the first time, showing the relevance of ethnopharmacology to characterize leishmanicidal molecules.

An ethanopharmacological study conducted among Chimane Indians from Amazonian Bolivia showed that stem bark *Pera benensis* Rusby has been used to treat cutaneous leishmaniasis caused by *L. (V.) braziliensis*. In the laboratory, it was verified that chloroform extracts containing quinones were active on promastigote forms of *L. (V.) braziliensis* (Fournet et al., 1992a). Further fractionation of the extract led to the identification of plumbagin, 3,3′-biplumbagin, 8-8′-biplumbagin and lupeol. Promastigote forms of *L. (L.) amazonensis, L. (V.) braziliensis* and *L. (L.) donovani* were eliminated when incubated with plumbagin; 3,3′-biplumbagin; 8-8′-biplumbagin; and intracellular amastigotes of *L. (L.) amazonensis* were highly sensitive to plumbagin and 3,3′-biplumbagin, which were able to eliminate 100 and 85% of intracellular parasites at 50 μg/ml.

Subsequently, an ethnopharmacological study conducted in Bolivia among settlers and Chimane Indians recorded that 14 plants were used to treat leishmaniasis as a topical poultice. Ten plants were indicated by the colonists and four by the indigenous people (Fournet et al., 1994). Extracts were prepared with different plant parts using petroleum ether, chloroform, and ethyl acetate of ethanol 50%; additionally, alkaloidal and quinoic fractions were also produced. Extracts were tested *in vitro* against *L*. *(L.) amazonensis*, *L. (V.) braziliensis,* and *L. (L.) donovani*; and from 10 plants indicated by the colonists, only *Bocconia integrifolia* Bonpl. and *B. pearcei* (Papaveraceae) were active. However, according to *Plants of the World online*, these plants are currently classified as synonyms, and the accepted name is *Bocconia integrifolia* Bonpl. Considering the four plants indicated by the Chimane Indians, extracts produced with the leaves, stem bark or root bark of the following three species were active on *Leishmania* *sp.*: *Galipea longiflora* K. Krause, *Ampelocera edentula* Kulm. and *Pera benensis* Rusby. In previous studies, it was demonstrated that 4-hydroxy-1-tetralone from *A. edentula*, three naphthoquinones from *P. benensis*, and quinoline alkaloids from *G. longiflora* displayed leishmanicidal activity (Fournet et al., 1989; 1992a; 1992b; 1993a). These studies reinforce that medicinal plants indicated by the Chimane Indians are potentially more effective than those indicated by the group of colonists, and extracts, fractions or purified molecules may be used as prototype drugs to treat human leishmaniasis according to the traditional knowledge of native people from Colombia.

An ethnopharmacological survey performed in northeastern Peru recorded 289 uses of plants for the treatment of leishmaniasis (Kvist et al., 2006). Twenty-eight plants were selected, and ethanolic extracts were produced and tested toward promastigote forms of *L. (L.) major*. It was observed that crude ethanolic extracts produced with the cortex of *Maytenus* *sp.*, *Minquartia guianensis, Aspidosperma rigidum,* with the roots of *Mansoa standleyi, Rauwolfia* *sp.*, *Tabernaemontana* *sp.*, with the bulb of *Curcuma longa* and with the resin of *Copaifera pauperi* displayed significant IC_50_ values against promastigote forms (between 10 and 20 μg/ml). In addition, 62 citations of the genus *Maytenus* were recorded in the treatment of leishmaniasis, suggesting that in addition to the high bioactivity of this plant on *L. (L.) major* (IC_50_ < 10 μg/ml), it has been used by different people living in traditional communities.

In the Yanesha community, Peru, ninety-four plants have been used to treat symptoms related to malaria and cutaneous leishmaniasis. In this community, twelve plants have been employed in the treatment of leishmaniasis (Valadeau et al., 2009); however, only eleven plants were tested in a laboratory context. In this case, ethanolic extracts of the plant parts were produced and assayed in axenic amastigote forms of *L. (L.) amazonensis*. Among plants used by the Yanesha group, ethanolic extracts produced from the leaves of *Carica papaya* L. (Caricaceae), *Hyptis lacustris* A. St.-Hil. ex Benth. (Lamiaceae) and *Lantana* *sp.* (Verbenaceae) were highly active plants for the elimination of parasites (IC_50_ = 10 μg/ml). However, it is important to note that the community uses the latex of *C. papaya*, the exudate of the bark or leaves from the stem from *Hyptis lacustris* and finally uses concentrated infusion of *Lantana* *sp.* This was the first study to record the leishmanicidal activity of latex from papaya (*C. papaya*). In addition, it was found that the treatment widely used in the fight against leishmaniasis by the community consists of the application of whitish latex, recently dripped from *Acalypha macrostachya* Jacq. (Euphorbiaceae) in the entire affected area for three consecutive days. This recipe is used for both cutaneous and mucocutaneous leishmaniasis. On the other hand, other plants used as traditional medicinal, such as *Vismia* *sp.* (Clusiaceae) and *Pityrogramma calomelanos* (L.) Link (Pteridaceae) showed low/moderate activity in the laboratory, possibly because the authors were unable to legitimately reproduce the mode of use, that is, testing the latex recently extracted from these plants, as indicated by the healers in the Yanesha community. On the other hand, some plants not employed in the traditional treatment of leishmaniasis also displayed significant leishmanicidal activity, as is the case for hydroalcoholic extracts produced with the leaves of *Cestrum racemosum* Ruiz & Pav. (IC_50_ = 9.8 μg/ml), *Piper dennisii* Trel. (IC_50_ = 10 μg/ml) and with the rhizome of *Hedychium coronarium* J. König (IC_50_ = 10 μg/ml), *Renealmia alpinia* (Rottb.) Maas (IC_50_ = 9 μg/ml) and *Renealmia thyrsoidea* (Ruiz & Pavon) Poepp. & Endl. (IC_50_ = 10 μg/ml).

In Colombia, an ethnopharmacological survey was carried out among Afro-Colombians and indigenous people to record plants traditionally used to treat malaria, Chagas disease and leishmaniasis. Based on ethnopharmacological and chemotaxonomy, the antiprotozoal activity of methylene chloride and methanolic extracts produced with 44 plants were analyzed. Among these plants, five have been used to treat leishmaniasis (Weniger et al., 2001). In this case, it was verified that the aerial parts of *Conobea scoparioides* (Scrophulariaceae) and *Hygrophila guianensis* (Acanthaceae), the bark exudate of *Otoba novogranatensis* and *O. parviflora* (Myristicaceae)*,* and *Castilla elastica* (Moraceae) have been used as traditional medicines to treat leishmaniasis. *In vitro* experiments showed that methylene chloride extract produced with the leaves of *C. scoparioides* was highly active at eliminating promastigote forms of *L. (L.) amazonensis, L. (L.) infantum* and *L. (V.) braziliensis*; additionally, macrophages infected with *L. (V.) panamensis* and incubated with this extract for 96 h eliminated 50% of parasites at 6.7 μg/ml. Methylene chloride and methanolic extracts produced with the fruits of *O. novagranatensis* were also active against the same species, and on amastigote forms, both eliminated intracellular *L. (V.) panamensis* (IC_50_ = 6.5 and 10.6 μg/ml, respectively). Apolar and polar extracts produced with the leaves of this plant also killed promastigote forms; however, they displayed only low or moderate activity on intracellular amastigotes (IC_50_ = 177 and >40 μg/ml, respectively); similar findings were observed with the apolar extract produced with the bark of *O. parvifolia*. Although some extracts displayed moderate or low activity on amastigote forms, once more, it becomes important to highlight the fundamental differences in the production of the natural medicines used by healers in communities and the way that researchers produce extracts in laboratories and use them in biological systems, which obviously minimizes the complexity of human physiology and the interactions between molecules, cells and parasites.

Table 1 summarizes the leishmanicidal activity of plants described above, displaying the 50% inhibitory concentrations (IC_50_) if available, parasite species and form (amastigote or promastigote) used and described in the selected articles.

### Contributions of some botanical families and species in the experimental treatment of leishmaniasis

In the present review, it was verified that at least 292 plants may be employed in the traditional treatment of leishmaniasis in different communities around the world, and it was verified that some families of plants have been widely used by communities, such as Apocynaceae, Araceae, Bignoniaceae, Asteraceae, Euphorbiaceae, Lamiaceae, Fabaceae, Malvaceae, Piperaceae, Rubiaceae, Rutaceae, Solanaceae and Verbenaceae. Below are described mainly *in vivo* studies about the efficacy of extracts and/or purified molecules from the botanical families used by traditional communities. Furthermore, details about the treatment, route of administration, parasite species, clinical form and efficacy of treatment are shown in Table 2.

Plants from the Apocynaceae family are rich in bioactive secondary metabolites (Siddiqui et al., 1986; Arambewela and Ranatunge, 1991; Muruganandam et al., 2000; Bhaskar and Natarajan, 2015; Kaunda and Zhang, 2017), and such molecules may have activity on tissue amastigote forms. In this regard, it was found that the genus *Tabernaemontana* has been cited several times in different communities as healing symptoms related to leishmaniasis, but few scientific advances have been made with this genus. Despite few works about the species traditionally used, it has been verified that the leishmanicidal effect of molecules purified from a related species, *T. catharinensis* A. DC., may be linked to the immunomodulatory activity of this genus (Soares et al., 2007). In addition, it was verified that the leishmanicidal molecule voacamine, an indole alkaloid purified from *T. divaricata* (L.) R.Br. ex Roem. & Schult, altered the mitochondria, kinetoplast and nucleus of *L. (L.) amazonensis* and *L. (L.) donovani* promastigotes, and such morphological changes correlated with the relaxation activity of topoisomerase IB. Additionally, it was verified that BALB/c mice infected with wild-type or drug-resistant *L. donovani* treated with 2.5 and 5 mg/kg voacamine by the intraperitoneal route twice a week for three weeks displayed fewer parasites in the spleen and liver than the untreated control (Chowdhury et al., 2017), reinforcing that this genus contains important classes of antileishmanial molecules. Although these species were not cited by traditional communities, it is possible that plants belonging to the same genus share similar compounds. Hexanic extract produced with the roots of the less cited species from this family, *Pentalinon andrieuxii* (Müll.Arg.) B.F.Hansen & Wunderlin, was active on promastigote forms of *L. (L.) mexicana in vitro* (Lezama-Dávila et al., 2007) and BALB/c mice infected with *L. (L.) mexicana* treated with 10 μg of this extract by the topical route, once a day for six weeks, presented fewer parasites in the skin; in addition, treated animals produced high levels of IL-12 cytokine along with the expression of the costimulatory molecules CD40, CD80, and CD86 (Lezama-Dávila et al., 2014), suggesting that, at least in part, the leishmanicidal activity *in vivo* may be associated with stimulation of innate immune cells. Further studies led to the identification of sterols from the roots of this plant that were active on intracellular amastigote forms of *L. (L.) mexicana* with an IC_50_ between 0.03 and 14.5 μM (Pan et al., 2012)*,* and the sterol pentalinonsterol encapsulated in liposomes, given by the intravenous route at 2.5 mg/kg, significantly reduced the number of viable parasites in the liver, spleen and bone marrow of BALB/c mice infected with *L. (L.) donovani*; additionally, this molecule activated the Th1 immune response in treated animals (Gupta et al., 2015). The genus *Aspidospermum* has been cited as a source of natural medicine against leishmaniasis, and bioactive alkaloids purified from different species of this genus may be responsible for the efficacy of plants observed in traditional communities (Tanaka et al., 2007; Reina et al., 2014); however, studies involving experimental models of leishmaniasis (*in vivo*) have not been performed thus far.

Different species of plants from the family Bignoniaceae were cited 13 times to treat symptoms associated with leishmaniasis in communities. Among these plants, it was demonstrated that the naphthoquinone lapachol, purified from *Handroanthus serratifolius* (Vahl) S.O.Grose, was active (*in vitro*) on amastigote forms of *L. (L.) amazonensis* (Costa et al., 2017)*,* and the possible mechanism of action of this molecule involves programmed cell death (Araújo et al., 2019)*.* In addition to the *in vitro* studies, it was demonstrated that lapachol, given orally for 10 days, decreased the number of amastigote forms of *L. (L.) amazonensis* in experimental cutaneous leishmaniasis, and a significant reduction in splenic and hepatic parasites was observed in visceral leishmaniasis caused by *L. (L.) infantum* (Araújo et al., 2019). In the same way, it was verified that *Jacaranda* species have also been traditionally used to treat leishmaniasis; however, only *in vitro* studies were carried out (Passero et al., 2007).

With respect to the family Asteraceae, 22 citations of plants that have been used in the context of skin diseases by traditional communities were observed. However, few works have been developed thus far with the most frequently cited genera. The genus *Munnozia*, cited as a healing agent, was studied with respect to leishmanicidal and tryponocidal activities. In this regard, the petroleum ether extract produced with the leaves of *Munnozia maronii* (André) H.Rob and the isolated compound dehydrozaluzanin C showed *in vitro* activity against *L. (L.) amazonensis*; additionally, it was demonstrated that dehydrozaluzanin C, given once a day for 14 days at 100 mg/kg, reduced the severity of cutaneous lesions in the experimental model of cutaneous leishmaniasis caused by *L. (L.) amazonensis* (Fournet et al., 1993b). Sesquiterpene lactones have also been isolated from *Pseudelephantopus spicatus* (Juss. ex Aubl.) C.F.Baker)*,* a species used by traditional communities, and the leishmanicidal activity of such molecules (IC_50_ = 0.2–0.99 μM) was similar to the activity of amphotericin B (IC_50_ = 0.41 μM), a second-line drug used in the treatment of patients with leishmaniasis (Odonne et al., 2011b). The thiophene derivative 5-methyl-2,2':5′,2″-terthiophene purified from *Porophyllum ruderale* (Jacq.) Cass. was also active on axenic amastigote forms of *L. (L.) amazonensis* (Takahashi et al., 2011), and such activity was associated with physiological and morphological alterations in parasite mitochondria (Takahashi et al., 2013). Despite these interesting *in vitro* data described with plants from the Asteraceae family that have been used by traditional communities, it was observed that experiments confirming the efficacy *in vivo* of molecules purified from plants used in traditional medicine are missing; however, *in vitro* data obtained with bioactive molecules suggest that plants produce and accumulate leishmanicidal compounds.

In the present review, 21 citations related to the traditional uses of plants from the Euphorbiaceae family were observed. Among them, the genus *Croton* has been used to treat skin diseases, and the medicinal activity can be related to the molecule linalool present in the essential oil of *Croton cajucara* Benth., which displayed a strong leishmanicidal potential against amastigote forms of *L. (L.) amazonensis* (IC_50_ = 8.7 ng/ml) and immunomodulatory effects on peritoneal macrophages that, once treated, were able to produce elevated amounts of nitric oxide, an important microbicidal molecule (Rosado et al., 2003). In addition, other compounds, such as 7-hydroxycalamenene, *trans*-dehydrocrotonin, *trans*-crotonin, and acetylaleuritolic acid, from *C. cajucara* Benth. also inhibited the proliferation of intracellular amastigote forms of *L. (L.) amazonensis* or *L (L.) chagasi* (Rosado et al., 2003; Rodrigues et al., 2013; Lima et al., 2015). Despite these phytochemical studies revealing the molecular diversity of the *Croton* genus as well as the leishmanicidal potential of molecules, only one study showed that a fraction purified from the hexanic extract from the leaves of *C. caudatus* Geiseler, given by oral route for five consecutive days at 5 mg/kg, reduced the number of viable parasites by 65 and 69% in the spleen and liver of experimental animals infected with *L. (L.) donovani*, respectively (Dey et al., 2015), and this therapeutic activity was associated with the restoration of IFN-γ levels in CD4 T lymphocytes. In addition to *Croton* species, several molecules purified from *Euphorbia* genus showed leishmanicidal activity *in vitro* on intracellular amastigotes, such as piceatannol, simiarenol, 1-hexacosanol, β-sitosterol, and β-sitosterol-3-*O*-glucoside (Duarte et al., 2008; Amin et al., 2017). Tannin- and saponin-rich fractions from the root of *E. wallichii* Hook.f. eliminated extra and intracellular forms of *L. tropica* with similar activities as the standard treatment; additionally, these fractions permeabilized the parasite’s cell membrane and triggered apoptosis in *L. tropica* (Ahmad et al., 2019), but to the best of our knowledge, no *in vivo* studies were performed with all of these purified molecules*.*

Traditional communities have used plants from the Fabaceae family to treat symptoms related to leishmaniasis. The genera *Copaifera, Desmodium, Lonchocarpus,* and *Senna* have been cited and recorded in different studies. The copaiba oil extracted from different species of *Copaifera* showed activity against promastigote and amastigote forms of *L. (L.) amazonensis* (Santos et al., 2008); additionally, it was observed that BALB/c mice infected with *L. (L.) amazonensis* and treated with the essential oil of *Copaifera martii* Hayne at 100 mg/kg by the oral, subcutaneous and topical routes displayed smaller skin lesions than untreated BALB/c mice (dos Santos et al., 2011). Further studies suggested that pinifolic and kaurenoic acids (Dos Santos et al., 2011) or β-caryophyllene may be responsible, at least in part, for the *in vitro* and *in vivo* activities observed in such studies. The species *Desmodium adscendens* and *D. axillare* have also been used as traditional remedies. Although scientific records about the leishmanicidal activity of such species do not exist, studies have already shown that the n-butanol fraction produced with whole *Pleurolobus gangeticus* (L.) J.St.-Hil. ex H.Ohashi & K.Ohashi plants given orally once a day for five consecutive days inhibited the multiplication of amastigote forms in the spleen of experimental animals with visceral leishmaniasis caused by *L. (L.) donovani* (Singh et al., 2005); on the other hand, the ethanolic extract and hexanic and aqueous fractions displayed moderate and weak leishmanicidal activity *in vivo*. Furthermore, the therapeutic activity of *D. gangeticum* may be associated with the occurrence of glycolipids, aminoglucosyl glycerolipids and cerebrosides in extracts (Mishra et al., 2005). Similarly, *Senna reticulata* is used by traditional communities, but pharmacological studies with respect to leishmanicidal activity have been performed only with *S. spectabilis* (DC.) H.S.Irwin & Barneby*,* and its activity was related to the presence of alkaloids (Melo et al., 2014), which can possibly interact with leishmanial arginase (Lacerda et al., 2018), inducing cell death; however, no proof of concept exists concerning the *in vivo* properties of such molecules.

Plants from the Piperaceae family have also been used by traditional communities, and there are many works addressing advances with the genus *Piper*. These works describe the molecular diversity of the genus as well as the leishmanicidal activity of the purified molecules. In this regard, it was observed that chalcones, phenolic compounds, lignans, and terpenes, among other molecules, display leishmanicidal properties (Torres-Santos et al., 1999; Hermoso et al., 2003; Cabanillas et al., 2010; Vendrametto et al., 2010; Garcia et al., 2013; Dal Picolo et al., 2014; Capello et al., 2015; Ceole et al., 2017). Additionally, it was observed that the possible cellular targets of such molecules were the mitochondria and plasma membrane of *Leishmania* *sp.* (Misra et al., 2009; de Oliveira et al., 2012), in addition, these molecules can stimulate immune responses, facilitating the destruction of intracellular parasites (Chouhan et al., 2015). Despite knowledge about the molecular diversity of the *Piper* genus and the bioactivity of such molecules on *Leishmania* *sp.,* only a few works have shown the *in vivo* relevance of this genus. Chalcone flavokavain B purified from the leaves of *Piper rusbyi* C. DC. given by the subcutaneous route to BALB/c mice infected with *L. (L.) amazonensis* at 5 mg/kg was able to reduce the size of lesions by 32% (Flores et al., 2007), and (E)-piplartine isolated from the leaves of *Piper pseudoarboreum* Yunck*,* given once a day for 4 days by the intralesional route at 25 mg/kg, reduced the size of cutaneous lesions by 35% and inhibited the visceralization of *L. (L.) amazonensis* in BALB/c mice (Ticona et al., 2020).

Plants from the Solanaceae family have been cited by traditional communities to treat symptoms related to leishmaniasis; however, only a few scientific advances have been made with plants of this family. Recently, it was demonstrated that hydroalcoholic extracts produced with the leaves of *Solanum havanense* Jacq.*, S. myriacanthum* Dunal*, S. nudum* Humb. & Bonpl. ex Dunal*,* and *S. seaforthianum* Andrews showed high selective indexes on *L. (L.) amazonensis* (*in vitro*) and in experimental leishmaniasis caused by *L. (L.) amazonensis,* it was observed that the hydroalcoholic extract produced with *S. havanense,* given every 4 days (5 doses) by the intralesional route at 30 mg/kg, decreased the number of parasites by 93.6%. Hydroalcoholic extracts produced with the leaves of *S. nudum, S. myriacanthum* and *S. seaforthianum* reduced the number of amastigotes in the skin of experimental animals by 80, 56.8 and 49.9%, respectively (Cos et al., 2018). In addition, it was demonstrated that the combination of the alkaloids solamargine and solasonine purified from *S. lycocarpum* A.St.-Hil. topically applied at 10 μg in the skin of C57BL/6 mice infected with *L. (L.) mexicana* reduced the size of cutaneous lesions and the number of tissue parasites (Lezama-Dávila et al., 2016), emphasizing the presence of potent bioactive molecules in the family Solanaceae.

Plants from the families Rubiaceae and Rutaceae have been used by traditional communities in the treatment of leishmaniasis; however, few works have characterized and tested the bioactive molecules of these plants (Muhammad et al., 2003; Quintin et al., 2009). Despite this, studies have shown that quinolines and alkaloids from *Angostura longiflora* (K.Krause) Kallunki (Rutaceae) exhibit leishmanicidal activity (*in vitro*), and *in vivo,* it was demonstrated that quinolic alkaloids from the bark or root of this plant given by oral or intralesional routes to experimental animals infected with *L. (L.) amazonensis* or *L. (V.) braziliensis* controlled the experimental infection, reducing the number of parasites in the skin (Fournet et al., 1996; Calla-Magariños et al., 2013); additionally, these studies suggested that animals treated by the intraperitoneal route displayed a significant reduction in parasites.

Some families were less cited by healers in communities; however, interesting results have been observed in the scientific literature, as is the case for *Dysphania ambrosioides* (Amaranthaceae) (L.) Mosyakin & Clemants. This plant has been used by a rural population in a coastal area of Bahia state, Brazil, in cases of cutaneous leishmaniasis (França et al., 1996). Experimentally, it was verified that the essential oil given by the intraperitoneal route once a day for 15 days at 30 mg/kg reduced the number of amastigote forms in the skin of BALB/c mice by 68% (Monzote et al., 2006). In addition, it was demonstrated that hydroalcoholic extract produced with the leaves of this plant given by the intralesional route reduced the number of amastigote forms of *L*. *(L.) amazonensis* in the skin, lymph nodes and spleen of BALB/c mice. However, the treatment given by the oral route did not alter the course of infection. The essential oil of *D. ambrosioides* (L.) Mosyakin & Clemants given by the oral route also reduced the number of amastigote forms in experimental cutaneous leishmaniasis caused by *L. (L.) amazonensis* (Patrício et al., 2008). Furthermore, it was demonstrated that the essential oil of this plant and its components can affect the mitochondria of parasites (Monzote et al., 2006, 2007; Pastor et al., 2015). *Allium sativum* L. (Amaryllidaceae), garlic, was cited only once as a traditional remedy for the treatment of leishmaniasis; however, advances concerning leishmanicidal activity *in vitro* and *in vivo* have been demonstrated. In the experimental model of cutaneous leishmaniasis caused by *L. (L.) major*, it was demonstrated that aqueous extract produced with dried bulbs of garlic, given by intraperitoneal route daily for 15 days at 20 mg/kg, inhibited the progression of cutaneous lesions as well as parasite multiplication. However, it was demonstrated that aqueous extract produced with fresh bulbs given at the same dose and route was inactive (Gamboa-León et al., 2007), but interestingly, it was verified that the aqueous extract produced with fresh bulbs of garlic collected in Hamadan (Iran), given by the intraperitoneal route at 20 mg/kg daily for 15 days to BALB/c mice infected with *L. (L.) major,* was able to reduce the size of lesions by 65% (Ghazanfari et al., 2000). These data suggest that the origin of garlic may impact the pharmacological activity of this plant. Methanolic extract produced with fresh bulbs and given daily by oral or intraperitoneal routes for 4 weeks also inhibited the size of cutaneous lesions in experimental animals infected with *L. (L.) major* by approximately 90 and 80%, respectively; and in experimental visceral leishmaniasis caused by *L. (L.) donovani,* the same treatment reduced the rate of parasitism in the spleen by 65 and 55% when it was given by oral or intraperitoneal routes, respectively (Wabwoba et al., 2010). Furthermore, the efficacy of *A. sativum* L. in leishmaniasis may be associated with the immunomodulatory activity of molecules produced by this plant (Ghazanfari et al., 2000; Gamboa-León et al., 2007). Unfortunately, no biomolecules were purified and assayed *in vivo* in an attempt to produce a standardized medicine.

*Maytenus* *sp.* (Celastraceae) has also been cited as a natural medicine used in leishmaniasis. It has been demonstrated that different species have leishmanicidal activity, and such activity can be mainly related to terpenes and sesquiterpenes synthesized by this genus (Alvarenga et al., 2008). Although only *in vitro* studies have been carried out so far, the most important finding is related to the potential of molecules against multidrug resistant parasites (Pérez-Victoria et al., 1999; Kennedy et al., 2001, 2011). The plant *Juniperus excelsa* M. Bieb (Cupressaceae) was cited only once by traditional communities, and few studies have been conducted on this species. The first published work showed that different extracts of the aforementioned species were able to eliminate *L. major* promastigotes (Moein et al., 2017). A further triple-blind randomized controlled clinical trial showed that 82% of patients with cutaneous leishmaniasis treated with a topical formulation produced with the leaves of *J. excelsa* M. Bieb hydroalcoholic extract plus cryotherapy healed the cutaneous lesions compared to the placebo control; additionally, they healed the lesions shorter than placebo control (Parvizi et al., 2017), suggesting that this plant species has bioactive molecules that can be further explored to develop new leishmanicidal drugs.

In this study, *Curcuma longa* L. (Zingiberaceae) was cited as a natural remedy for leishmaniasis only once. However, the leishmanicidal activity of curcumins has been recorded since 2000 (Rasmussen et al., 2000; Saleheen et al., 2002), and further works demonstrated that synthetic derivatives also present high activity at eliminating extra- and intracellular parasites (Gomes et al., 2002; Chauhan et al., 2018; Teles et al., 2019), and such activity may be related to programmed cell death in *L. donovani* (Chauhan et al., 2018). Despite these advances, *in vivo* studies with *Curcuma longa* L. or curcumin are scarce in the literature.

In addition, it was verified that the species *Urtica dioica* L. (Urticaceae) was cited only once, and just one work was published characterizing the leishmanicidal activity of this plant. In this regard, BALB/c mice infected with *L. major* and treated with the aqueous extract of *E. dioica* L. at 150, 200 or 250 mg/kg by intralesional or intramuscular routes three times per week for 30 days significantly decreased the size of cutaneous lesions and suppressed the dissemination of parasites to the spleen; furthermore, the *in vivo* activity was related to the reduction of arginase levels (Badirzadeh et al., 2020). This enzyme is able to inhibit nitric oxide production, and therefore, low levels of this circulating enzyme may be essential to achieve cure in leishmaniasis.

Details about families, plant species, clinical form of leishmaniasis, parasite species, extract or purified molecules employed in the treatment, doses, route of administration, scheme of treatment and efficacy of the treatments in experimental leishmaniasis are shown in Table 2.

## Limitations

In the present review, it was observed that only 20 articles addressed the traditional treatment of leishmaniasis using medicinal plants. Despite the few articles published to date, a substantial diversity of plants (89 plant families referring to 292 plants) has been cited by 29 traditional communities from different nationalities, which in fact supports the local treatment of symptoms related to leishmaniasis. On the other hand, this potential is far from reflecting reality, and there is still considerable work from an ethnopharmacological point of view to be conducted, which will certainly expand our knowledge about medicinal plants with antileishmanial properties. In this review, the authors emphasize that future ethnopharmacological studies must follow methodological rigor, consistent with the data to be collected. This should be carefully considered because in this review, several limits were found in terms of analysis due to the unavailability of some ethnopharmacological data in the articles consulted. As examples, only 74% of the plants were identified to the species level, 36.5% specified the recipes, 20.6% detailed the route of administration, and only 55.5% mentioned the vernacular names of the plants. Furthermore, 12.9% of the articles did not mention the community that provided traditional knowledge, and some of the authors referred to them as local people or ethnic groups. This is a critical point in the field of ethnopharmacology, as it weakens the right to intellectual property of the traditional communities involved. Furthermore, it was observed that practically no article mentioned the contraindications and possible adverse reactions to these plants, although it is well known that traditional communities often obtain this knowledge from their therapeutic practices. These specific data would be relevant in the case of the development of drugs to treat leishmaniasis, since it is necessary to find drugs with fewer adverse reactions in comparison with those currently in use.

In addition, although a plethora of plants have been described in the traditional treatment of leishmaniasis, only a few works were capable of describing them from a chemical or pharmacological point of view. Furthermore, only a minority of them analysed, in experimental models of cutaneous or visceral leishmaniasis, the efficacy of such plants or purified molecules. Finally, it would be promising to perform bioprospective studies on such plants, since in fieldwork, researchers have already observed their curative properties, which in fact could shorten the time of development of an effective medicine.

## Future Perspectives and Priorities

This review opens up a huge range of research possibilities in the field of leishmaniasis from a chemical and pharmacological point of view. Table 1 presents 292 plants (216 species and 76 genera) to be investigated as extracts and/or as drugs aimed at developing antileishmanial medicines. Some of these possible “hint plants” are presented in *Contributions of Some Botanical Families and Species in the Experimental Treatment of Leishmaniasis*. The botanical families and genera that had a higher frequency of citations during this survey are presented and compared with data from other studies in this section.

In addition, the species most frequently mentioned in articles and by the traditional communities in certain countries were highlighted throughout the text. In this context, four species are noteworthy since they were mentioned in four articles: *Carica papaya* L. (Caricaceae), *Cedrela odorata* L. (Meliaceae), *Copaifera paupera* (Herzog) Dwyer (Fabaceae), and *Musa × paradisiaca* L. (Musaceae), while *Nicotiana tabacum* L. (Solanaceae), *Carica papaya* L. (Caricaceae), and *Musa × paradisiaca* L. (Musaceae) were cited simultaneously by traditional communities from Peru, Ecuador, and French Guiana. Thus, these last two species are among the most cited in articles and by traditional communities.

On the other hand, it becomes important to note that the majority of articles dealing with extracts or purified molecules from plants with ethnopharmacology relevance presented only an inhibitory concentration of 50% against promastigote and/or amastigote forms. Although such data shed light on this scenario, articles should investigate the leishmanicidal properties of plant extracts or molecules against the intracellular amastigote form, which is the form of the parasite that persists and causes disease in the host. Furthermore, it was observed that preclinical studies with medicinal plants traditionally used to treat leishmaniasis are surprisingly rare, but they should be encouraged, since the proof of concept—that a given plant has therapeutic activity in humans—was already provided by healers, and in these specific cases, scientists should standardize mandatory steps related to phytochemistry, pharmacology and parasitology to produce effective medicines.

Finally, this review suggests that future investigations should be guided but not limited to the five species cited above, expanding the chance of discovering new medicines for this disease since, according to the survey presented herein, few or no studies have been performed with plants traditionally used to treat leishmaniasis.
